# Host-Microbe-Drug-Nutrient Screen Identifies Bacterial Effectors of Metformin Therapy

**DOI:** 10.1016/j.cell.2019.08.003

**Published:** 2019-09-05

**Authors:** Rosina Pryor, Povilas Norvaisas, Georgios Marinos, Lena Best, Louise B. Thingholm, Leonor M. Quintaneiro, Wouter De Haes, Daniela Esser, Silvio Waschina, Celia Lujan, Reuben L. Smith, Timothy A. Scott, Daniel Martinez-Martinez, Orla Woodward, Kevin Bryson, Matthias Laudes, Wolfgang Lieb, Riekelt H. Houtkooper, Andre Franke, Liesbet Temmerman, Ivana Bjedov, Helena M. Cochemé, Christoph Kaleta, Filipe Cabreiro

**Affiliations:** 1 MRC London Institute of Medical Sciences, Du Cane Road, London W12 0NN, UK; 2 Institute of Clinical Sciences, Imperial College London, Hammersmith Hospital Campus, Du Cane Road, London W12 0NN, UK; 3 Institute of Structural and Molecular Biology, University College London and Birkbeck, London WC1E 6BT, UK; 4 Institute for Experimental Medicine, Kiel University, 24105 Kiel, Germany; 5 Institute of Clinical Molecular Biology, Christian Albrechts University of Kiel, 24105 Kiel, Germany; 6 Molecular and Functional Neurobiology, Department of Biology, KU Leuven, 3000 Leuven, Belgium; 7 UCL Cancer Institute, University College London, London WC1E 6JD, UK; 8 Laboratory of Genetic Metabolic Diseases, Amsterdam UMC, University of Amsterdam, 1105 AZ Amsterdam, the Netherlands; 9 Department of Computer Science, University College London, London WC1E 6BT, UK; 10 Department of Internal Medicine I, University Hospital Schleswig-Holstein, Campus Kiel, Kiel, Germany; 11 Institute of Epidemiology, Christian Albrechts University Kiel, 24105 Kiel, Germany

**Keywords:** aging, *C. elegans*, *Drosophila*, humans, type-2 diabetes, metabolic modeling, CRP signaling, metformin, microbiome, diet

## Abstract

Metformin is the first-line therapy for treating type 2 diabetes and a promising anti-aging drug. We set out to address the fundamental question of how gut microbes and nutrition, key regulators of host physiology, affect the effects of metformin. Combining two tractable genetic models, the bacterium *E. coli* and the nematode *C. elegans*, we developed a high-throughput four-way screen to define the underlying host-microbe-drug-nutrient interactions. We show that microbes integrate cues from metformin and the diet through the phosphotransferase signaling pathway that converges on the transcriptional regulator Crp. A detailed experimental characterization of metformin effects downstream of Crp in combination with metabolic modeling of the microbiota in metformin-treated type 2 diabetic patients predicts the production of microbial agmatine, a regulator of metformin effects on host lipid metabolism and lifespan. Our high-throughput screening platform paves the way for identifying exploitable drug-nutrient-microbiome interactions to improve host health and longevity through targeted microbiome therapies.

**Video Abstract:**

## Highlights

• A high-throughput method for investigating host-microbe-drug-nutrient interactions
• Metformin host effects are regulated by a bacterial nutrient signaling pathway
• Metabolic modeling of human gut microbiomes links metformin to microbial agmatine
• Metformin-bacterial interactions engage host lipid metabolism to extend lifespan

## Graphical Abstract

**Figure:**
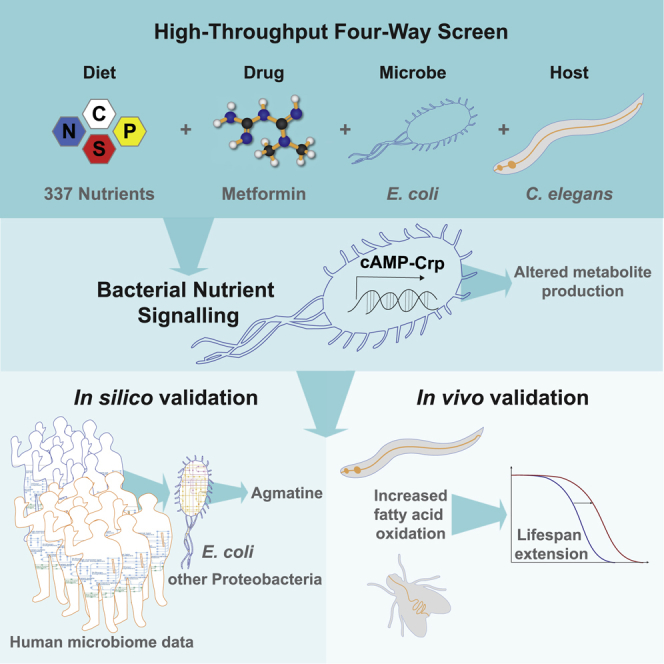

## Teaser

Looking at the effects of diet, microbiome, and host biology on drug responsiveness highlights pathways contributing to metformin’s effects on lifespan.

## Introduction

The microbiota is widely acknowledged as a central regulator of host health (Kundu et al., 2017, Schmidt et al., 2018). Environmental cues, including drugs and diet, drive changes in microbial ecology and function (Maier et al., 2018, Rothschild et al., 2018) with important consequences for host health. However, the causal dynamics controlling these interactions are largely unknown. The biguanide metformin, a putative dietary restriction mimetic (Pryor and Cabreiro, 2015), is the most widely prescribed drug for type 2 diabetes. Unexpectedly, metformin treatment increases the survival of type 2 diabetic patients compared with matched healthy controls (Barzilai et al., 2016). The effects of metformin on host physiology are regulated by its interaction with the microbiota in an evolutionarily conserved manner, from *C. elegans* to humans (Bauer et al., 2018, Cabreiro et al., 2013, Forslund et al., 2015, Wu et al., 2017). For example, metformin treatment does not extend *C. elegans* lifespan in the absence of bacteria, when bacteria are metabolically impaired, or when bacteria develop resistance to the growth-inhibitory effects of metformin (Cabreiro et al., 2013). Nutrition also plays a key role in regulating both host and microbial physiology (David et al., 2014) as well as the efficacy of drugs in treating disease (Gonzalez et al., 2018). Indeed, the effects of metformin on host physiology are dependent on dietary intake (Bauer et al., 2018, Shin et al., 2014). However, the precise mechanisms by which microbes regulate these effects in a nutrient-dependent manner remain elusive.

Given the complexity of microbial metabolism and the myriad of metabolites of prokaryotic origin regulating host-related processes, understanding and harnessing their potential is a challenging task. Like humans, *C. elegans* hosts a community of gut microbes that acts as a central regulator of host physiology (Zhang et al., 2017). Recently, microbial metabolites of interest have been identified using animal models that allow direct high-throughput measurements of quantifiable and conserved host phenotypes that are directly regulated by microbes (Qi and Han, 2018). Moreover, similar to the human microbiota, *C. elegans* is dominantly colonized by enterobacteria (Lloyd-Price et al., 2017, Zhang et al., 2017), making it an ideal model for studying the effect of human gut microbes such as *E. coli* on host physiology and their function in mediating the response to host-targeted drugs (Cabreiro et al., 2013, Garcia-Gonzalez et al., 2017, Scott et al., 2017). Although many efforts have been made to develop techniques that further our understanding of the role of microbial genetics in host regulation, none exist to dissect the intricate relationships between nutrition, pharmacology, microbes, and host physiology.

Here we devise a high-throughput four-way screening approach to facilitate the evaluation of nutritional modulation of drug action in the context of the host-microbe meta-organism. Using this strategy, we identify a bacterial signaling pathway that integrates metformin and nutrient signals to alter metabolite production by the microbiota. Changes in metabolite production can, in turn, affect fatty acid metabolism in the host, altering the lifespan. Importantly, using a computational modeling approach, we show that these changes in metabolite production are also recapitulated in the microbiota of metformin-treated type 2 diabetic patients, providing a potential explanation for the pro-longevity effects of metformin in humans.

## Results

### Four-Way Host-Microbe-Drug-Nutrient Screens Identify a Signaling Hub for the Integration of Drug and Nutrient Signals

**Figure 1:**
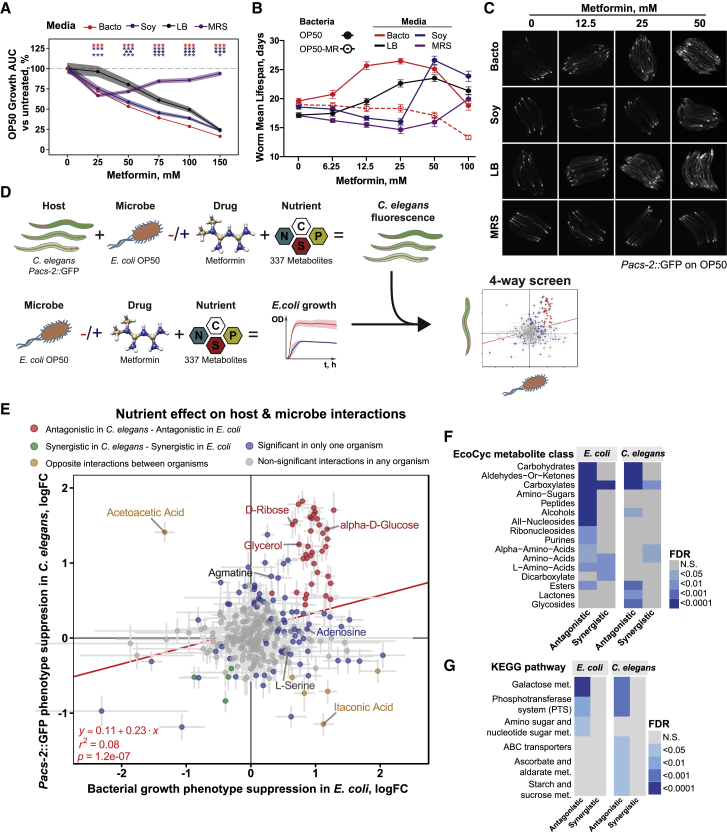
Four-Way Host-Microbe-Drug-Nutrient Screens Identify a Signaling Hub for the Integration of Drug and Nutrient Signals (A–C) The effects of metformin on bacterial growth (A), wild-type N2 worm lifespan (B), and metabolism (C) are dependent on drug dose, nutrients, and bacteria. OP50-MR is an *E. coli* OP50 strain that developed metformin resistance. As observed previously (Cabreiro et al., 2013), metformin does not extend the lifespan when worms are grown on OP50-MR. In (B), each data point corresponds to the mean lifespan of 80–154 worms. See also Table S1. In (C), each panel shows 8 individual worms. (D) Diagram of the four-way host-microbe-drug-nutrient interaction screen. (E) Nutrient effects on bacterial phenotype (growth, x axis) and on wild-type N2 worm phenotype rescue (*Pacs-2*::GFP expression, y axis) in response to metformin. The red fit line shows the correlation between metformin and nutrient effects in bacteria and worms. Antagonistic or synergistic refers to the type of interaction determined by linear modeling observed between metformin and nutrient effects, leading to an overall effect that is significantly greater than the sum of the effects of the two components alone either in *C. elegans Pacs-2*::GFP levels or *E. coli* growth. Positive fold changes indicate nutrient suppression of the effect of metformin in bacterial growth or *C. elegans Pacs-2*::GFP expression. Error bars represent SE. FDR < 0.05 for significance. All colored circles are statistically significant. Gray circles are non-significant. Effects of highlighted nutrients are provided in detail in Figures S1 and S2. (F and G) EcoCyc metabolite class (F) and KEGG pathway (G) enrichment for the effects of nutrients on *E. coli* OP50 growth and worm *Pacs-2*::GFP expression in the context of metformin treatment. Data are represented as mean ± SEM. ^∗^p < 0.05, ^∗∗^p < 0.01, ^∗∗∗^p < 0.001. See also Table S1 for lifespan statistics and Table S2 for screen statistics.

**Figure S1:**
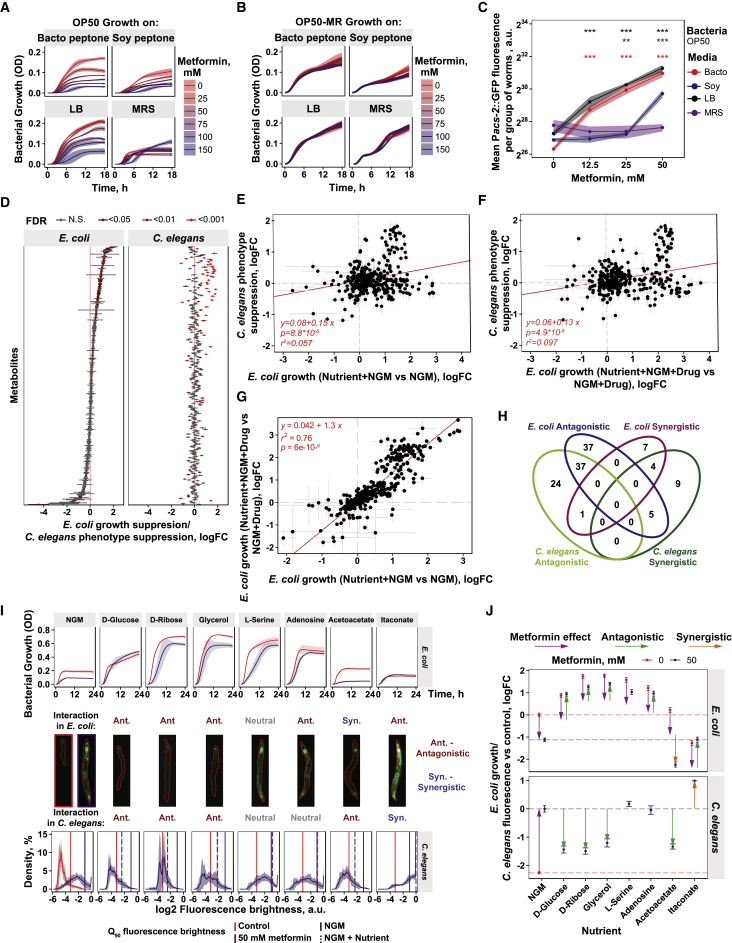
Four-Way Host-Microbe-Drug-Nutrient Screens Identify a Signaling Hub for the Integration of Drug and Nutrient Signals, Related to Figure 1 (A) Bacterial growth of *E. coli* OP50 on different types of media with increasing concentrations of metformin. Shaded area shows mean growth OD ± SD. (B) Bacterial growth of *E. coli* OP50-MR (metformin resistant) on different types of media with increasing concentrations of metformin. Shaded area shows mean growth OD ± SD. (C) *Pacs-2*::GFP expression of worms grown on *E. coli* OP50 with different types of media and increasing concentrations of metformin. Significance stars represent comparison with 0 mM metformin for each media type. (D) Comparison of nutrient effects on *E. coli* OP50 growth and worm *Pacs-2*::GFP expression in the context of metformin treatment. (E and F) Correlation between nutrient rescue of worm *Pacs-2*::GFP fluorescence and nutrient effect on *E. coli* OP50 growth in control (E), and metformin treatment conditions (F). Nutrient supplementation without metformin (r^2^ = 0.057, p = 8.8 × 10^−6^) (E) nor nutrient supplementation with metformin (r^2^ = 0.097, p = 4.9 × 10^−9^) (F) does strongly predict the effects of metformin on host physiology. (G) Strong correlation (r^2^ = 0.76, p = 6.0 × 10^−6^) between effects of nutrient supplementation on *E. coli* OP50 growth in control (x axis) versus metformin treatment conditions (y axis). (H) Venn diagram of nutrients with significant effects on *E. coli* and/or worms in the context of metformin treatment. (I) Top panel: Bacterial growth curves on base NGM media and with nutrient supplementation. Shaded area represents mean growth OD ± SD. Here and in following panels, red corresponds to control and purple to metformin treatment conditions. Middle panel: Examples of worm *Pacs-2*::GFP expression with the corresponding nutrient supplementation and the type of drug-nutrient interaction in worm response. Bottom panel: Histograms of worm *Pacs-2*::GFP expression in log_2_ scale, with distribution density shown on y axis. Shaded area shows worm brightness distribution SD for individual worms. Vertical lines indicate Q_90_ worm *Pacs-2*::GFP expression values. Red- Control and Blue – 50 mM metformin. Full lines- NGM control and dotted lines – NGM plus indicated nutrient supplementation. Full lines are represented in all conditions as a reference for direct comparison. (J) Bacterial growth estimates based on log_2_ transformed AUC values (top) and worm *Pacs-2*::GFP expression estimates based on log_2_ transformed fluorescence brightness Q_90_ values (bottom). Dashed lines indicate bacterial growth on NGM and a worm *Pacs-2*::GFP expression level used as a reference. Arrows indicate metformin treatment and significant interaction effects (FDR < 0.05).

We hypothesized that changing the nutritional context might alter the effects of metformin on bacterial growth and, in turn, modulate the metabolic and longevity response of *C. elegans* to metformin. Because metformin induces a dietary restriction-like state in *C. elegans* to regulate the organismal lifespan (Onken and Driscoll, 2010), we used the transgenic reporter *C. elegans* strain *Pacs-2*::GFP (Burkewitz et al., 2015), whose expression is an indicator of the transcriptional response under conditions of dietary restriction, to test this hypothesis. *Acs-2* is an acyl-coenzyme A (CoA) synthase ortholog that mediates the activation of fatty acids for β-oxidation in response to dietary restriction. As predicted, the ability of metformin to impair bacterial growth (Figures 1A, S1A, and S1B), enhance host longevity (Figure 1B; Table S1), and increase the expression of *Pacs-2*::GFP (Figures 1C and S1C) varied dramatically according to metformin concentration. Critically, the magnitude of these effects differs depending on the growth medium, suggesting a nutritional input into this response (Figures 1B and 1C).

To investigate how specific nutrients affect metformin action on the host in a bacterium-dependent way, we developed a high-throughput four-way host-microbe-drug-nutrient screen that allowed us to map these interactions at an extensive scale (Figure 1D; see STAR Methods for details). Briefly, we determined the propensity of 337 specific nutrients to modify the effect of metformin on bacterial growth. This provides a simple readout of nutrient-metformin interactions at the bacterial level. Similarly, measuring the expression levels of the *Pacs-2*::GFP *C. elegans* reporter line, we determined the propensity of these 337 nutrients to modify the dietary restriction-like transcriptional and metabolic response in the host induced by metformin in the presence of bacteria. This allowed us to identify nutrients that act in the host in the context of metformin in a bacterium-dependent and -independent manner (Figures 1D, 1E, and S1D; Table S2). Fold change values in the metformin-dependent *Pacs-2*::GFP fluorescence response of worms in the presence of specific nutrients (y axis) were plotted relative to the bacterial growth fold change values (x axis) for the same condition (Figure 1E). Although *E. coli* growth and *C. elegans* phenotype rescue by nutrients were not fully predictive of each other (r^2^ = 0.08, p = 1.1 × 10^−7^; Figures 1E and S1E–S1G), a large subset of the nutrient-bacterium interactions strongly predicted the effects of metformin on host physiology (Figure 1E, red circles). We observed that 37 of 79 nutrients that significantly rescued metformin-induced impairment of bacterial growth also suppressed *Pacs-2*::GFP activation by metformin in worms, and 25 nutrients that suppressed *Pacs-2*::GFP activation by metformin had either a neutral or synergistic interaction with the effects of metformin on bacterial growth (Figure S1H). Taken together, these data suggest specific nutritional tuning of metformin effects on host metabolism through the bacteria.

**Figure S2:**
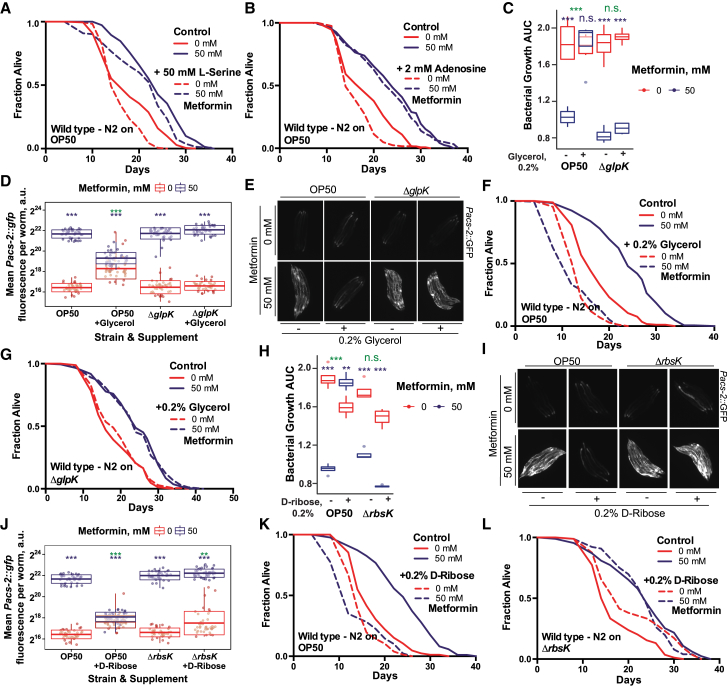
*E. coli* Integrates Drug and Nutritional Cues to Regulate Host Physiology, Related to Figure 1 (A and B) Supplementation with L-serine (A) or adenosine (B) does not suppress worm lifespan extension by metformin. (C) Supplementation with glycerol rescues inhibition of bacterial growth by metformin in control *E. coli* OP50 but not in OP50 Δ*glpK* mutants unable to catabolize glycerol. (D–G) Glycerol supplementation suppresses metformin-induced upregulation of *Pacs-2::*GFP expression (D-E) and abolishes lifespan extension (F-G) in worms in a bacteria-dependent manner. Nutrient effects are rescued by an *E. coli* OP50 Δ*glpK* mutant unable to catabolize glycerol. In (E), each panel shows 5 individual worms. (H) Supplementation with D-ribose rescues inhibition of bacterial growth by metformin in control *E. coli* OP50 but not in OP50 Δ*rbsK* mutants unable to catabolize D-ribose. (I–L) D-ribose supplementation suppresses metformin-induced upregulation of *Pacs-2::*GFP expression (I-J) and abolishes lifespan extension (K-L) in worms in a bacteria-dependent manner. Nutrient effects are rescued by an *E. coli* OP50 Δ*rbsK* mutant unable to catabolize D-ribose. In (I), each panel shows 5 individual worms. Data are represented as mean ± SEM unless otherwise stated. ^∗^p < 0.05; ^∗∗^p < 0.01; ^∗∗∗^p < 0.001. For C, D, H, and J, significance stars represent metformin effect (purple) and metformin-nutrient interaction (green). See also Table S1 for lifespan statistics and table S2 for screen statistics.

Next we performed a nutrient EcoCyc metabolite class enrichment analysis on both the bacterial growth and *Pacs-2*::GFP host data (Figure 1F; Table S2) with the aim to identify nutrients that specifically rescue metformin effects on host physiology through the bacteria. From our metabolite enrichment analysis, nutrients belonging to the classes of amino sugars, peptides, amino acids (e.g., L-serine), and nucleotides (e.g., adenosine) significantly rescued the effects of metformin on bacterial growth without affecting the effects of metformin on host metabolism and lifespan (Figures 1E, 1F, S1I, S1J, S2A, and S2B). Conversely, carbohydrates, aldehydes, or carboxylates (e.g., D-glucose, D-ribose, and glycerol) rescued *E. coli* growth and abolished both the upregulation of *Pacs-2*::GFP and lifespan extension in worms in a bacterium-dependent manner, as demonstrated by the specific deletion of bacterial genes responsible for nutrient catabolism (Figures 1E, 1F, S1I, S1J, and S2C–S2L). This suggests the presence of specific processes in bacteria integrating the effects of nutrients and metformin to regulate host physiology. To identify these processes, we performed Kyoto Encyclopedia of Genes and Genomes (KEGG) ontology pathway analysis for both *E. coli* and *C. elegans* (Figure 1G; Table S2). This analysis revealed enrichment for the galactose and the phosphotransferase system (PTS) as key metabolic and signaling bacterial pathways, respectively, mediating the effects of metformin on the host. Altogether, our findings suggest a mechanism whereby the presence of specific metabolic and signaling pathways in bacteria function to integrate signals from both nutrition and drugs to regulate host metabolism.

### Bacterial Proteomics Identify Crp and ArgR as Transcriptional Regulators of Metformin Effects

**Figure 2:**
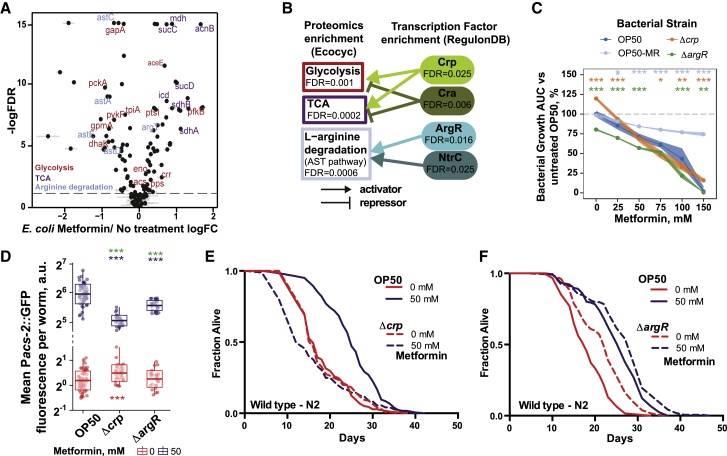
Bacterial Proteomics Identify Transcriptional Networks Underlying Metformin Effects in *E. coli* (A) Volcano plot showing *E. coli* proteins that are differentially regulated in response to metformin. Highlighted proteins belong to significantly enriched KEGG pathways. (B) Diagram displaying connectivity between KEGG pathway enrichment and RegulonDB transcription factor (TF) enrichment from proteomics data of *E. coli* OP50 treated with metformin. (C) Bacterial growth summary of *E. coli* OP50 TF mutants with metformin. Significance stars represent comparison with OP50 for each metformin concentration. (D) Metformin regulates worm *Pacs-2*::GFP expression in a bacterial TF-dependent manner. Significance stars represent comparison with OP50 at 0 mM (red) or 50 mM (purple) and metformin-genotype interaction (green). (E and F) Metformin extends worm lifespan in a bacterial TF-dependent manner. Data are represented as mean ± SEM. ^∗^p < 0.05, ^∗∗^p < 0.01, ^∗∗∗^p < 0.001. See also Table S1 for lifespan statistics and Table S3 for proteomics statistics.

To better understand the mechanistic links between the metabolic and signaling pathways identified in our four-way screen and the regulation of host physiology, we performed proteomics analyses of *E. coli* with and without metformin treatment (Figure 2A). Metformin treatment was significantly associated with specific KEGG pathways, such as upregulation of the tricarboxylic acid (TCA) cycle (false discovery rate [FDR] = 0.0002) and downregulation of both glycolysis (FDR = 0.001) and arginine degradation via the arginine N-succinyltransferase (AST) pathway (FDR = 0.0006) (Figure 2B; Table S3). We also performed a functional analysis of these proteome changes using the *E. coli* gene-transcription factor links from the RegulonDB database to identify signaling regulators underlying these functional changes in the context of metformin. Eleven transcription factors (TFs) were found to be significantly associated with the bacterial response to metformin (STAR Methods; Table S3). Only four remained statistically significant following multiple comparisons adjustment: Crp (FDR = 0.025), Cra (FDR = 0.006), ArgR (FDR = 0.016), and NtrC (FDR = 0.025) (Figure 2B).

**Figure S3:**
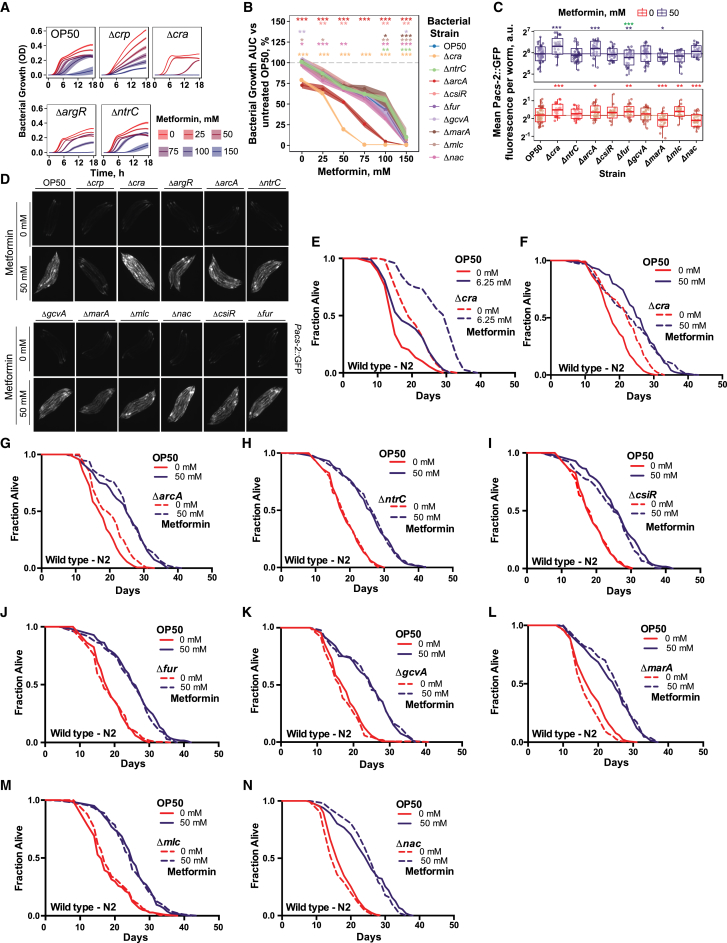
Bacterial Proteomics Identify Transcriptional Networks Underlying Metformin Effects in *E. coli*, Related to Figure 2 (A) Bacterial growth curves of *E. coli* OP50 transcription factor (TF) mutants with increasing concentrations of metformin. Shaded area shows mean growth OD ± SD. (B) Bacterial growth summaries of *E. coli* OP50 deletion mutants for TFs associated with proteomic changes in response to metformin treatment. Significance stars represent comparison with OP50 for each metformin concentration. Opposite to the effects of metformin on the resistant OP50-MR strain compared to OP50, Δ*cra* and Δ*arcA* mutants exhibited increased sensitivity to bacterial growth inhibition by metformin. (C and D) Metformin regulates worm *Pacs-2*::GFP expression in a *E. coli* OP50 TF-dependent manner. Worms grown on Δ*cra* (A) and Δ*arcA* mutants (B) showed an increased activation of host *Pacs-2*::GFP expression in an additive manner to metformin. For C, significance stars represent comparison with OP50 at 0 mM (red) or 50 mM (purple) and metformin-genotype interaction (green). In (D), each panel shows 5 individual worms. (E and F) Worm lifespan extension by metformin is enhanced with a *Δcra E. coli* OP50 mutant at low (6. 25 mM) (E) but not high (50 mM) (F) drug concentrations. As previously reported (Cabreiro et al., 2013), these data suggest a shift in the window of action of metformin on host longevity depending on the sensitivity of the bacterial strain to growth inhibition by metformin. (G-N) Survival curves of *E. coli* OP50 TF mutants that do not affect worm lifespan extension by metformin. Data are represented as mean ± SEM unless otherwise stated. ^∗^p < 0.05; ^∗∗^p < 0.01; ^∗∗∗^p < 0.001. See also Table S1 for lifespan statistics and Table S3 for proteomics statistics.

We investigated the role of these 11 bacterial TFs in regulating the effects of metformin on bacterial growth, host metabolism, and lifespan. Deletion of the bacterial TFs did not confer resistance to metformin (Figures 2C; S3A, and S3B). Analysis of the effect of these bacterial TFs on host metabolism (Figures 2D; S3C, and S3D) and lifespan (Figures 2E, 2F, and S3E–S3N) revealed that deletion of bacterial Crp and ArgR significantly reduced the upregulation of worm *Pacs-2*::GFP (Figures 2D). In addition, bacterial Crp and ArgR are fully and partially required, respectively, for the increased host longevity induced by metformin (Figures 2E and 2F). Together, these data suggest that a bacterial signaling mechanism mediates the effects of metformin on host metabolism and lifespan.

Therefore, we identified transcriptional regulators of metformin-*E. coli* effects on host physiology—a master regulator of carbon metabolism, Crp, and a master regulator of nitrogen metabolism, ArgR (Chubukov et al., 2014)—through a mechanism that is independent of bacterial resistance to the effects of metformin on growth.

### *E. coli* PTS-Crp Signaling Integrates Metformin and Nutrient Effects on Host Lifespan

**Figure 3:**
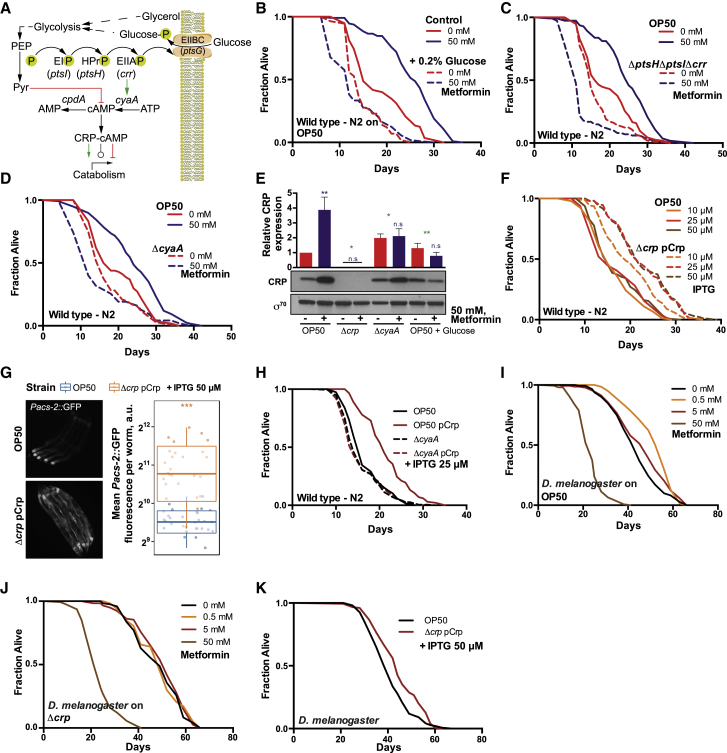
Bacterial PTS-Crp Signaling Regulates Metformin Effects on Host Metabolism and Lifespan (A) Diagram of the PTS-Crp signaling pathway in *E. coli*. (B–D) Glucose supplementation (B); deletion of *E. coli* OP50 *pts H*, *I,* and *crr* (C); and *cyaA* (D) abolishes worm lifespan extension by metformin. (E) Metformin upregulates Crp expression in control *E. coli* OP50 but not in OP50 *Δcrp* or *ΔcyaA* mutants or with glucose supplementation. Significance stars represent metformin effect (purple) and metformin-genotype or nutrient interaction (green). (F) Dose-dependent upregulation of Crp in *E. coli* OP50 extends the worm lifespan. (G) Overexpression of Crp in *E. coli* OP50 upregulates *Pacs-2::*GFP expression in worms. Each panel shows 5 individual worms. (H) Effect of overexpression of *E. coli* Crp on the worm lifespan is dependent on bacterial *cyaA*. (I and J) Metformin extends the lifespan in flies grown on chemically defined medium with *E. coli* OP50 (I) but not with an OP50 *Δcrp* mutant (J). (K) *E. coli* OP50 overexpressing Crp extends the fly lifespan on chemically defined medium. Data are represented as mean ± SEM. n.s., non-significant; ^∗^p < 0.05; ^∗∗^p < 0.01; ^∗∗∗^p < 0.001. See also Table S1 for lifespan statistics.

**Figure S4:**
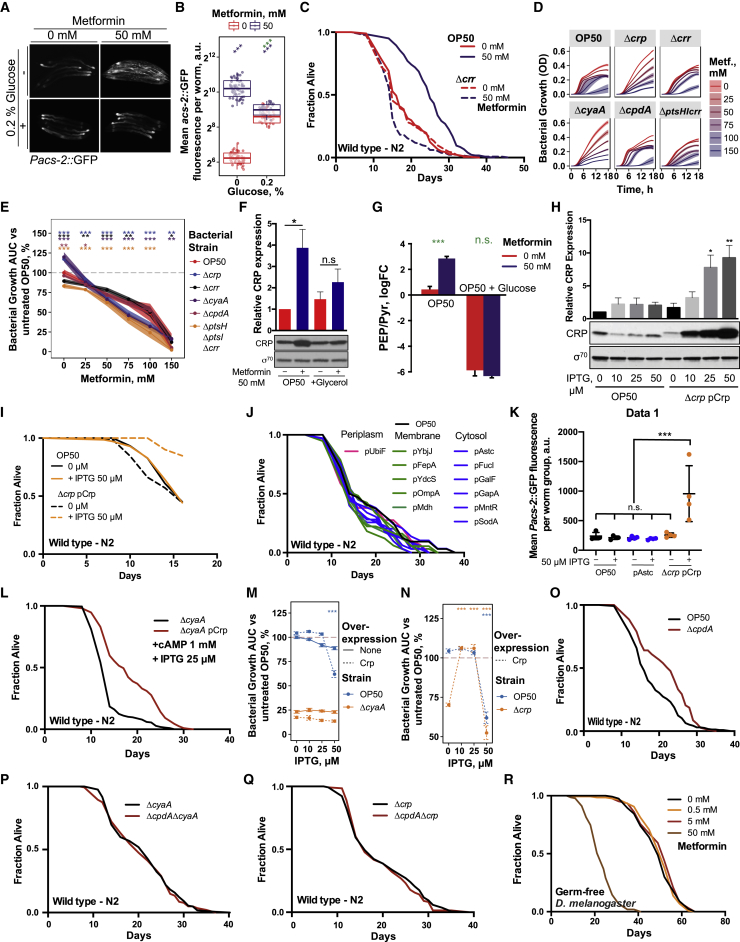
Bacterial PTS-Crp Signaling Regulates Metformin Effects on Organismal Metabolism and Lifespan, Related to Figure 3 (A and B) Glucose supplementation suppresses upregulation of worm *Pacs-2::*GFP expression by metformin. For B, significance stars represent metformin effect (purple) and metformin-nutrient interaction (green). In (A), each panel shows 5 individual worms. (C) Deletion of *E. coli* OP50 *crr* abolishes worm lifespan extension by metformin. (D) Bacterial growth curves of *E. coli* OP50 PTS-Crp signaling mutants with increasing concentrations of metformin. Shaded area shows mean growth OD ± SD. (E) Bacterial growth summaries of *E. coli* OP50 PTS-Crp signaling mutants with increasing concentrations of metformin. Significance stars represent comparison with OP50 for each metformin concentration. (F) Glycerol supplementation suppresses upregulation of Crp in metformin-treated *E. coli* OP50. (G) Metformin significantly increases the ratio of PEP/Pyruvate, the glycolytic flux sensor, in *E. coli* but the effect is abolished by glucose supplementation. (H) An *E. coli* OP50 *Δcrp* pCrp strain exhibits augmented Crp expression in response to increasing concentrations of IPTG. (I) Induction of PCrp overexpression is required to extend *C. elegans* lifespan. IPTG supplementation at 50 μM does not extend worm lifespan. (J) Overexpression of functionally diverse *E. coli* proteins in distinct sub-cellular compartments does not extend *C. elegans* lifespan implying that overexpression alone by a protein-inducible plasmid in bacteria does not affect *C elegans* lifespan. (K) Induction of *E. coli* pCrp overexpression is required to increase P*acs-2*:*:*GFP expression in worms. (L) Worms grown on *ΔcyaA* pCrp *E. coli* OP50 are longer lived compared to worms grown on *ΔcyaA E. coli* OP50 when supplemented with cAMP (1 mM) and 25 μM IPTG. (M) Growth summaries of OP50 and *Δcrp E. coli* OP50 strains overexpressing Crp in response to increasing concentrations of IPTG. Significance stars represent comparison with 0 μM IPTG for each strain. (N) Growth summary of OP50 and *ΔcyaA E. coli* OP50 strains overexpressing Crp in response to increasing concentrations of IPTG. Significance stars represent interaction between Crp overexpression and IPTG versus untreated control. (O–Q) An *E. coli* OP50 *ΔcpdA* mutant unable to degrade cAMP extends worm lifespan (K) but not in the absence of *cyaA* (L) and *crp* (M). (R) Metformin does not extend lifespan of germ-free flies in chemically-defined media. Data are represented as mean ± SEM unless otherwise stated. n.s. non-significant, ^∗^p < 0.05; ^∗∗^p < 0.01; ^∗∗∗^p < 0.001. See also Table S1 for lifespan statistics.

Our four-way screen combined with our proteomics approach identified the bacterial PTS-Crp axis as a central regulator of metformin effects on the host. The PTS is a major active transport system in bacteria that coordinates the uptake of multiple carbohydrate molecules with the downstream regulation of Crp via a cascade of phosphorylation events. Consequently, Crp, together with its binding partner cyclic AMP (cAMP), directly controls the transcription of hundreds of genes in response to the nutritional environment and adjusts metabolic processes accordingly (Chubukov et al., 2014; Figure 3A). We therefore conducted interventions designed to interrupt this signaling pathway at various steps. As expected, supplementation with glucose, a known inhibitor of bacterial cAMP-CRP signaling, reduced the effects of metformin on both the activation of *Pacs-2*::GFP in the host (Figures S4A and S4B) and lifespan extension (Figure 3B). Similarly, the use of bacterial mutants with deletions of multiple (Δ*ptsH*Δ*ptsI*Δ*crr*) or single (Δ*crr*) PTS proteins or adenylate cyclase (Δ*cyaA*) also abolished the effects of metformin on longevity (Figures 3C, 3D, and S4C). All mutant bacterial strains tested were equally or more sensitive to metformin (Figures S4D and S4E). This further supports that this bacterial signaling pathway regulates the host lifespan in response to metformin via its downstream effects on metabolism rather than by conferring direct resistance to the drug, as shown previously for OP50-MR (Cabreiro et al., 2013).

Next we tested whether metformin altered the expression levels of Crp. Metformin increased Crp expression in control bacteria but not in Δ*crp* or Δ*cyaA* mutant bacteria, nor in bacteria supplemented with glucose (Figure 3E) or the non-PTS sugar glycerol (Figure S4F). Altogether, this suggests cAMP-dependent upregulation of Crp linked to altered central carbon metabolism flux (Chubukov et al., 2014, You et al., 2013). Consistent with this, the ratio of the flux sensor phosphoenolpyruvate (PEP)/pyruvate, whose levels are known to regulate Crp activation through a PTS-CyaA signaling mechanism (You et al., 2013), were increased by 533% under the metformin condition (p = 0.0002) and abolished by glucose supplementation (p = 0.338; Figure S4G; Table S4). Therefore, we asked whether we could mimic the metformin effects on host metabolism by genetically activating Crp signaling in *E. coli*. This was achieved through control of Crp levels under an isopropyl-β-D-thiogalactoside (IPTG)-inducible promoter (Figure S4H). Crp overexpression in *E. coli* extended the lifespan in a dose-dependent manner (Figures 3F, S4I, and S4J) and upregulated host *Pacs-2*::GFP (Figures 3G and S4K). In addition, consistent with the role of cAMP in Crp activation, Crp overexpression required CyaA and cAMP to increase the worm lifespan (Figures 3H and S4L), an effect independent of loss of bacterial fitness because of overexpression (Figures S4M and S4N). Similarly, deletion of the cAMP-degrading enzyme *cpdA* increased the worm lifespan in a *cyaA-* and *crp*-dependent manner (Figures S4O–S4Q). Our data suggest that activation of the functional signaling unit requires both the TF and its cofactor to promote effects on host health.

To determine whether this phenomenon is also present in other species, we investigated the effects of metformin on *Drosophila* lifespan. Although metformin extends the lifespan in multiple organisms, it has been shown previously that it failed to extend the lifespan of *Drosophila* (Slack et al., 2012). However, using a fully chemically defined medium whose composition was based on nutritional findings from our four-way screen (STAR Methods), we showed that metformin does extend the *Drosophila* lifespan in a dose-dependent manner when colonized with control OP50 *E. coli* (Figure 3I) but not in germ-free flies (Figure S4R) or those colonized with a Δ*crp* mutant (Figure 3J). Overexpressing Crp in *E. coli* was sufficient to increase the *Drosophila* lifespan (Figure 3K), further highlighting the evolutionary conservation of this bacterial pathway in regulating the host lifespan. Overall, these findings demonstrate that the overexpression of bacterial Crp elicits similar effects as metformin on the host, implying a common overlapping mechanism.

### Bacterium-Derived Agmatine Underlies Metformin Effects on Host Metabolism and Lifespan

**Figure 4:**
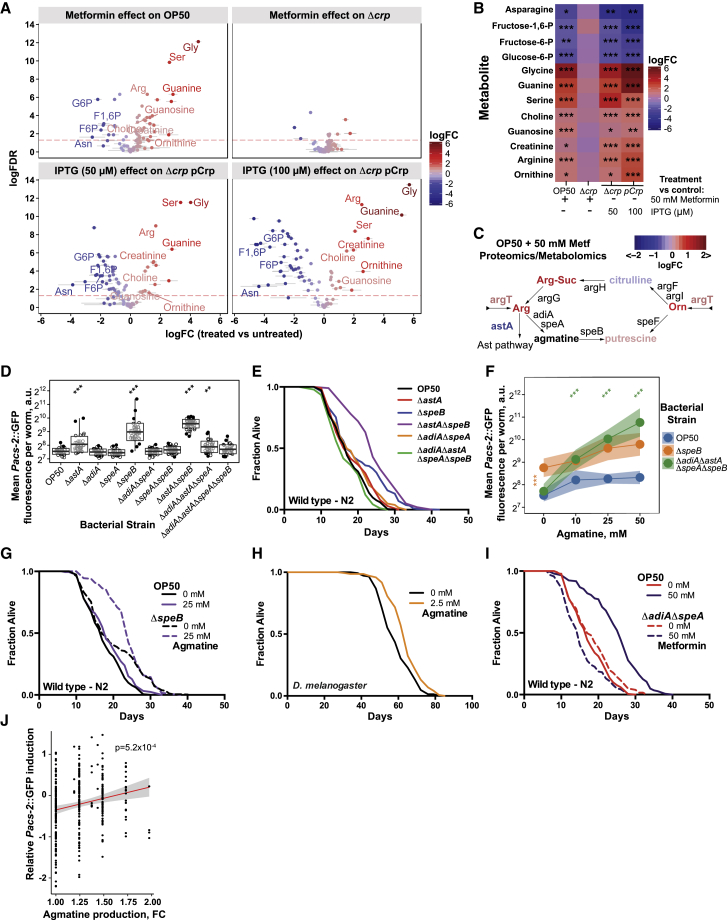
Bacterial Agmatine Regulates Host Metabolism and Lifespan (A) Volcano plots of metabolomics data showing effect of metformin in control *E. coli* OP50 or an OP50 *Δcrp* mutant and the effect of Crp overexpression in OP50. (B) Subset of differentially and significantly expressed metabolites that are unique to Crp regulation and metformin treatment. (C) Bacterial arginine-related metabolic pathways with an overlay of metformin-induced changes in the *E. coli* proteome and metabolome. Ast, arginine N-succinyltransferase pathway. (D and E) Deletion of genes from *E. coli* arginine catabolism alters worm *Pacs-2*::GFP expression (D) and lifespan (E). (F and G) Agmatine supplementation upregulates worm *Pacs-2*::GFP expression (F) and extends the lifespan (G) in a bacterium-dependent manner. (H) Agmatine supplementation extends the fly lifespan in sugar-yeast-agar (SYA) medium. (I) Metformin does not extend the lifespan in the agmatine-deficient OP50 mutant *Δadi*A*ΔspeA*. (J) Comparison of *in silico* predicted agmatine production capacity and measured worm *Pacs-2*::GFP expression with nutrient supplementation in the context of metformin. The p values indicate the significance of association between predicted agmatine production capacity and *Pacs-2*::GFP fluorescence (linear model fit). See Figure S5J for predicted agmatine production capacity and measured growth-rescue of metformin-treated *E. coli* OP50. Data are represented as mean ± SEM. ^∗^p < 0.05, ^∗∗^p < 0.01, ^∗∗∗^p < 0.001. See also Table S1 for lifespan statistics, Table S3 for proteomics statistics, and Table S4 for metabolomics statistics.

**Figure S5:**
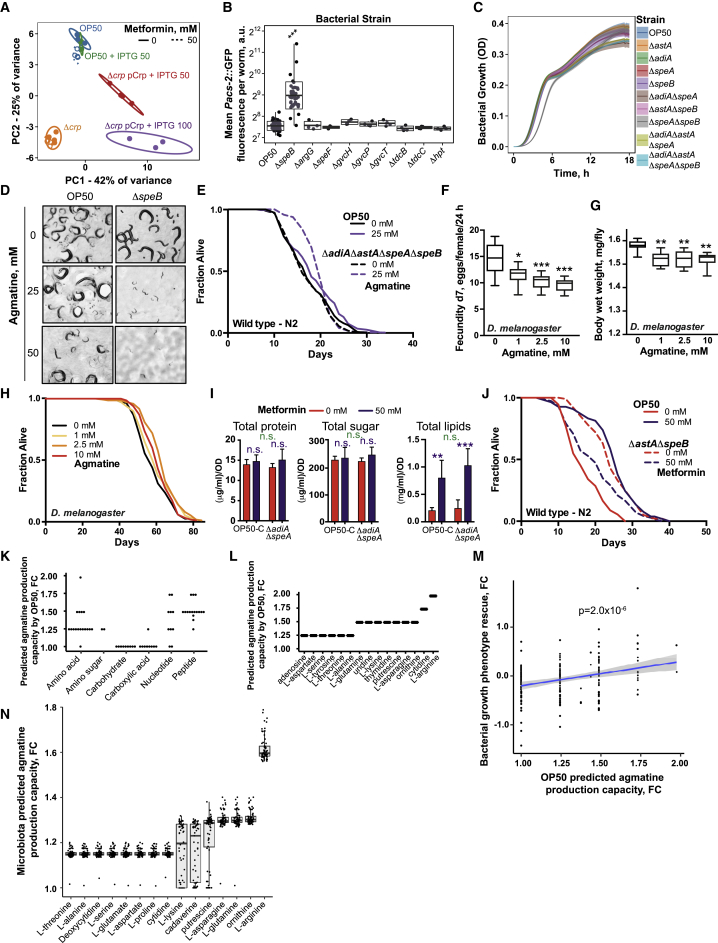
Bacterial Agmatine Regulates Host Metabolism and Lifespan, Related to Figure 4 (A) PCA plot of *E. coli* metabolomics data showing effect of metformin treatment on control *E. coli* OP50 and a OP50 *Δcrp* mutant and the effect of Crp overexpression. (B) Worm *Pacs-2*::GFP expression is increased by a *ΔspeB E. coli* OP50 mutant. (C) Bacterial growth curves of *E. coli* arginine catabolism mutants. Shaded area show mean growth OD ± SD. (D) Agmatine supplementation delays worm development and reproduction in a bacteria-dependent manner. (E) Agmatine supplementation extends lifespan in worms grown on a *ΔadiAΔastAΔspeAΔspeB E. coli* OP50 mutant unable to metabolize agmatine. (F–H) Agmatine supplementation reduces *Drosophila* fecundity (F) and body weight (G) and extends *Drosophila* lifespan (H) in a concentration-dependent manner on SYA media. (I) Measurements of macromolecular content (proteins, sugars and lipids) of control *E. coli* OP50 and a *ΔadiAΔspeA* OP50 mutant show no significant differences between the strains. Significance stars represent metformin effect (purple) and metformin-genotype interaction (green). (J) Metformin does not extend lifespan further when worms are grown on a *ΔastAΔspeB E. coli* OP50 mutant. (K) Predicted relative increase in agmatine production by *E. coli* OP50 following supplementation of 5 mmol of different nutrients to NGM medium. Nutrients are grouped according to their class. (L) Top 15 metabolites according to predicted increase of agmatine production by *E. coli* OP50 on NGM medium following supplementation of 5 mmol of each compound. Only compounds present in the diet of the Kiel cohort are shown. (M) Comparison of predicted increases in agmatine production following nutrient supplementation to NGM medium and experimentally measured *E. coli* OP50 growth phenotype rescue by nutrients on Biolog plates in response to metformin. A significant association between predicted agmatine production capacity and measured growth-rescue of metformin-treated *E. coli* OP50 (linear model p = 2.0 × 10^−6^, Table S5D). (N) Predicted increases in agmatine production capacity of the microbiota of metformin-treated patients following supplementation 1 mmol of each compound to the reported diet of the participant available per gram of microbiota. Only compounds present in the diet of the Kiel cohort are shown. Abbreviations: FC, fold-change. Data are represented as mean ± SEM unless otherwise stated. n.s. non-significant, ^∗^p < 0.05; ^∗∗^p < 0.01; ^∗∗∗^p < 0.001. Abbreviations: FC, fold-change. See also Table S1 for lifespan statistics and Table S4 for metabolomics statistics.

Crp regulates a myriad of metabolic processes in bacteria (Chubukov et al., 2014). To understand the Crp-dependent metabolic changes in *E. coli* relevant to host lifespan, we used an *E. coli* metabolomics approach to identify metabolite level changes that were common to both metformin-treated OP50 and Crp overexpression but absent in the metformin-treated Δ*crp* strain (Figures 4A, 4B, and S5A; Table S4). Volcano plots showed dramatic differences between the metabolic profiles of OP50 and Δ*crp* treated with metformin, suggesting that Crp strongly influences the metabolic response of *E. coli* to metformin (Figure 4A).

To test the importance of accumulated bacterial metabolites in regulating host physiology, we created *E. coli* mutants with deletions of genes known to utilize these metabolic substrates and to be under the regulation of Crp (Figure S5B). Of these gene deletion strains, only a strain with a mutation in the *speB* gene, which catabolizes agmatine (Figure 4C), conferred a significant increase in host *Pacs-2*::GFP expression. We therefore focused on arginine catabolism because (1) deletion of *speB* (agmatinase) or its repression by Crp in the absence of sugars impairs arginine catabolism via the agmatinase pathway, leading to an accumulation of agmatine (Satishchandran and Boyle, 1986; Figure 4C); (2) arginine degradation via an alternative route known as the AST pathway was strongly downregulated by metformin treatment (Figures 2A and 2B; Table S3); and (3) several metabolites from arginine metabolism were significantly altered in *E. coli* treated with metformin (Figure 4C; Table S4). As expected, single- or double-deletion mutants in both arginine catabolism pathways (Δ*astA*, Δ*speB*, and Δ*astA*Δ*speB*), which are predicted to accumulate agmatine, induced host *Pacs-2*::GFP expression. A quadruple mutant, Δ*adiA*Δ*astA*Δ*speA*Δ*speB*, which was not expected to accumulate agmatine, did not upregulate *Pacs-2*::GFP, implying that bacterial agmatine rather than arginine regulates host metabolism (Figure 4D). The host lifespan was similarly affected by these mutant strains (Figure 4E). Given that we observed no significant loss of growth fitness in these bacterial mutants (Figure S5C), ruling out confounding effects, our data suggest a direct link between bacterial agmatine production and host metabolism and longevity.

We further investigated the role of agmatine by exogenous supplementation. Agmatine delayed worm development and reproductive output (Figure S5D), upregulated *Pacs-2*::GFP expression (Figure 4F), and increased the lifespan (Figures 4G and S5E). As expected, the effect on worm physiology was more significant when grown on bacterial mutants that cannot metabolize agmatine (i.e., Δ*speB* and Δ*adiA*Δ*astA*Δ*speA*Δ*speB*) (Figures 4G and S5E). Likewise, agmatine reduced *Drosophila* fecundity (Figure S5F) and weight (Figure S5G) and increased the lifespan (Figures 4H and S5H) in a dose-dependent manner, suggesting evolutionary conservation of agmatine effects on host physiology. Next we tested the effects of metformin when worms were grown on Δ*adiA*Δ*speA* mutants, which cannot produce agmatine, and found that metformin no longer extended the worm lifespan (Figure 4I). We did not observe any differences in macromolecular nutrient content between OP50 and the Δ*adiA*Δ*speA* mutant (Figure S5I), suggesting that it is agmatine, rather than other nutritional changes induced by metformin, that drives the lifespan effects on the host. Finally, when worms were grown on a bacterial Δ*astA*Δ*speB* mutant strain that maximally accumulated agmatine, metformin did not further extend their lifespan (Figure S5J). Taken together, these data strongly support a model in which agmatine, rather than other nutritional changes induced by metformin in bacteria, drives the lifespan effects on the host.

Next we used a metabolic model of *E. coli* OP50 (Zimmermann et al., 2019) to determine the effect of nutrient supplementation on agmatine production capacity (Figures S5K and S5L; Tables S5C and S5D). The metabolic model predicts that sugars do not increase bacterial agmatine production capacity, whereas nucleotides, amino acids, and peptides do (Figures S5K and S5L; Table S5B). This is consistent with data obtained from the four-way screen, as exemplified by a significant association of predicted changes in agmatine production upon nutrient supplementation with *Pacs-2*::GFP induction in *C. elegans* (Figure 4J; linear model p = 5.2 × 10^−4^; Table S5C). The predicted increase in agmatine production capacity showed a discrete stepwise clustering (Figures S5K and S5L) that could be explained by the number of nitrogen residues gained by *E. coli* OP50 during degradation of the correspondent nutrient (Table S5), which is reflected in the high nitrogen content of agmatine. Thus, the *in silico* model predicts that many metabolites identified in our four-way screen mediate their effect through increased agmatine production by *E. coli* in a metformin-dependent manner. Overall, these data provide a causal link between metformin supplementation and agmatine production by bacteria to increase the host lifespan.

### The Microbiota of Metformin-Treated Patients Has Increased Agmatine Production Capacity

**Figure 5:**
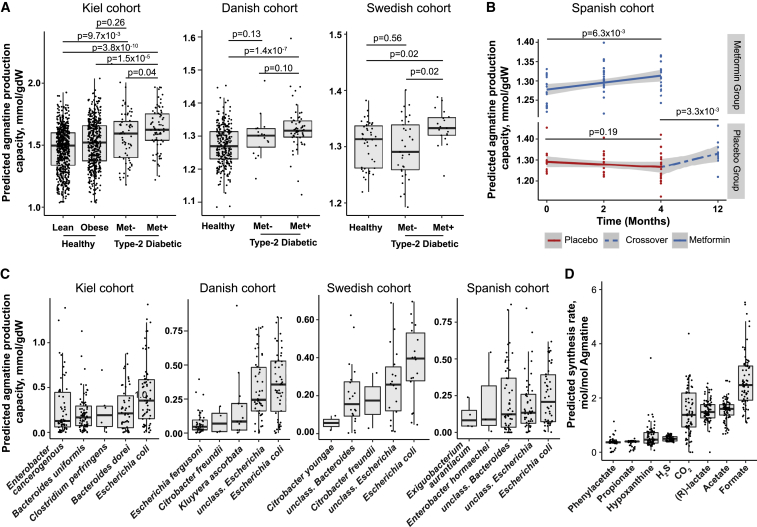
Metabolic Modeling of Human Gut Microbiota Reveals Signatures of Agmatine Overproduction in Metformin-Treated Type 2 Diabetic Patients (A) Predicted agmatine production by the gut microbiota in the 3 independent cohorts. Shown are FDR-corrected p values from Wilcoxon rank-sum tests between the indicated groups. (B) Longitudinal changes in predicted agmatine production following initiation of metformin treatment in newly diagnosed type 2 diabetic patients. The p values indicate the significance of the treatment effect (i.e., time) on agmatine production (linear model fit). (C) Predicted top 5 microbial producers of agmatine within the gut microbiome of metformin-treated patients across cohorts. (D) Side products of predicted agmatine production in the Kiel cohort. Values correspond to moles of side product produced per mole of agmatine produced. Data are represented as absolute values. For details regarding statistical tests, see STAR Methods and Table S5. mmol/gM/day, predicted production fluxes in millimoles per gram of gut microbiota per day.

To establish whether there is a link between bacterial agmatine production and metformin treatment in humans, we investigated whether metformin treatment is associated with increased agmatine production capacity in the microbiota of metformin-treated type 2 diabetic patients (Kiel cohort; STAR Methods), using microbial community modeling (Graspeuntner et al., 2019, Magnúsdóttir et al., 2017) specifically accounting for the dietary intake of each patient (STAR Methods). In this modeling approach, 16S rRNA sequencing data are mapped to a repository of metabolic models of bacteria of the gut microbiome (Magnúsdóttir et al., 2017). Subsequently, these metabolic models are joined together into a metabolic microbial community model that accounts for the abundance of individual bacterial species and is constrained by the dietary uptake of each participant. By using linear optimization on these models, the agmatine production capacity of each participant’s microbiome can be predicted (STAR Methods). Within the Kiel cohort, comprising 1,258 human participants (Table S5E), the predicted agmatine production capacity was significantly higher in metformin-treated type 2 diabetic patients (n = 76; Figure 5A) than in untreated type 2 diabetic patients (n = 57, FDR = 0.04), healthy obese controls (n = 492, FDR = 1.5 × 10^−5^), and healthy lean controls (n = 633, FDR = 3.8 × 10^−10^; Table S5G). Untreated type 2 diabetic patients showed no difference in agmatine production capacity compared with healthy obese controls (FDR = 0.26) and only a small increase compared with healthy lean controls (FDR = 9.7 × 10^−3^). Similar results were observed for a Swedish and a Danish cohort (Figure 5A; Tables S5I and S5J). Consistent with our *in silico* nutrient supplementation screen in *E. coli* OP50, we also observed the strongest increases in predicted agmatine production capacity of human gut microbial communities following supplementation of nitrogen-rich compounds in the Kiel cohort (Figure S5N; Table S5B). Moreover, we tested the influence of different phenotypic variables on the predicted agmatine production capacity in the Kiel cohort. Even when controlling for body mass index and age, agmatine production remained most strongly associated with metformin-treatment status (STAR Methods; Table S5H).

To fully exclude phenotypic differences as a confounder causing differences in agmatine production capacity between groups, we next assessed agmatine production capacity in a longitudinal cohort (Wu et al., 2017; see STAR Methods for cohort setup). Metformin treatment was associated with a significant increase in agmatine production capacity both after initiation of metformin treatment and after switching a placebo group to metformin (p = 6.3 × 10^−3^ and p = 3.3 × 10^−3^, respectively), with no significant effect with placebo alone (p = 0.19) (Figure 5B; Table S5K). Modeling results revealed that the strongest producers of agmatine were bacteria from the genera *Escherichia*, *Bacteroides*, *Enterobacter*, and *Citrobacter* (Figure 5C), which are consistently more abundant in metformin-treated patients across cohorts (Forslund et al., 2015, Wu et al., 2017; Table S5L). Additionally, we determined fermentation products such as the short-chain fatty acids acetate and propionate as well as CO_2_ and H_2_S (Figure 5D) as major side products of agmatine synthesis.

Overall, the modeling data informed by our four-way screen support the conclusion that metformin interactions with the microbiota promote the production of agmatine (in a nutrient-dependent manner) and, as a direct consequence, other metabolites that may contribute to metformin’s beneficial action (e.g., short-chain fatty acids) as well as its negative side effects such as bloating and other gastrointestinal complications (e.g., through production of CO_2_ and H_2_S) (Forslund et al., 2015, Pryor and Cabreiro, 2015, Wu et al., 2017).

### Bacterium-Mediated Increases in Host Fatty Acid Oxidation Extend the Host Lifespan

**Figure 6:**
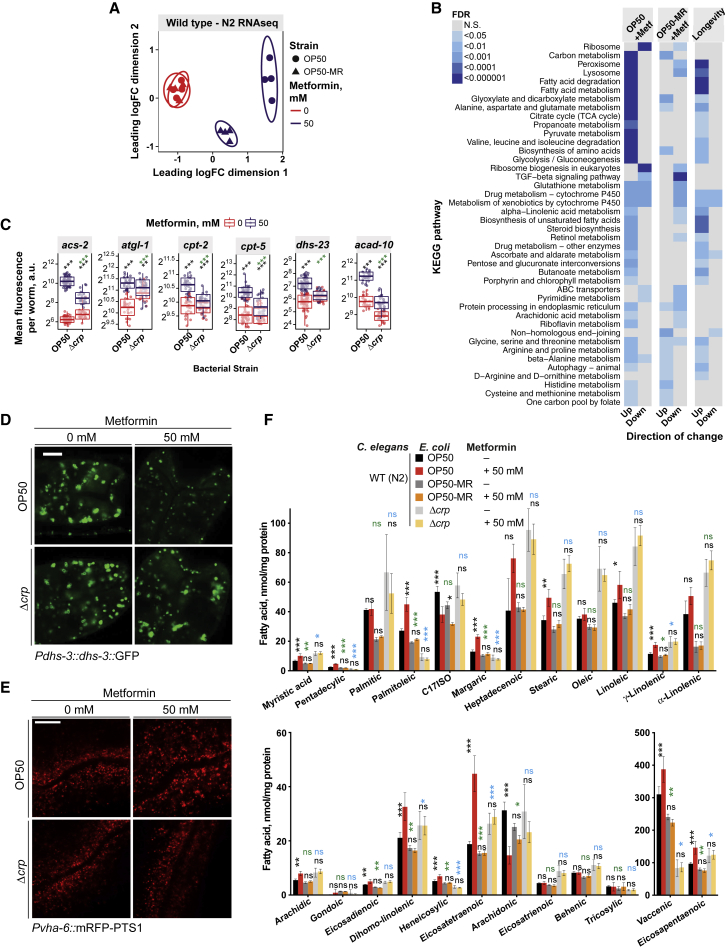
Metformin and Bacterium-Dependent Transcriptional and Metabolic Signatures in *C. elegans* (A) Multi-dimensional scaling plot of worm RNA-seq data showing distinct and bacterium-dependent transcriptional signatures associated with metformin treatment. (B) KEGG pathway enrichment for worm RNA-seq data. (C) Metformin increases expression of worm lipid-related genes in a bacterium-dependent manner as effects are suppressed in OP50 *Δcrp*. Similar effects were observed for worms grown on OP50-MR (Figures S6B and S6C). (D and E) Confocal visualization of worm lipid droplets (D) and peroxisomes (E), showing effects of metformin in worms in a bacterial Crp-dependent manner. Similar effects were observed for worms grown on OP50-MR (Figures S6E and S6F). Scale bars, 10 μm. No changes in gene expression for *dhs-3* or *vha-6* were observed (Table S6). (F) Metabolomics in worms show an interaction between metformin and bacteria on host fatty acid profiles. Data are represented as mean ± SEM. ^∗^p < 0.05, ^∗∗^p < 0.01, ^∗∗∗^p < 0.001. In (E) and (F), significance stars represent metformin effect (black) and metformin-bacterium interaction (green or blue). See also Table S6 for RNA-seq statistics and Table S7 for fatty acid metabolomics statistics.

**Figure S6:**
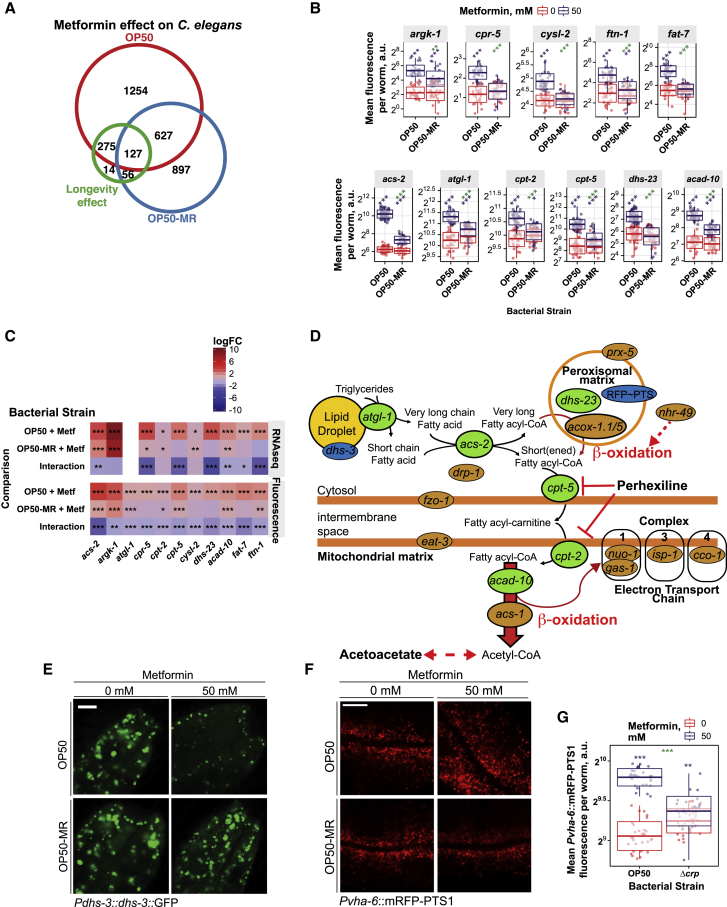
Metformin and Bacterium-Dependent Transcriptional and Metabolic Signatures in *C. elegans*, Related to Figure 6 (A) Venn diagram showing an overlap of metformin-induced significant (FDR < 0.05) transcriptional changes in worms on *E. coli* OP50 and OP50-MR strains, and the subset responsible for the longevity phenotype. (B) Metformin increases the expression of worm genes involved in multiple processes in a bacteria-dependent manner. Significance stars represent metformin effect (purple) and metformin-bacteria interaction (green). (C) Metformin-induced increases in worm gene expression revealed by RNaseq are recapitulated using fluorescent transgenic reporter lines. (D) Diagram of genes and metabolites involved in fatty acid metabolism that were studied in order to evaluate their contribution to metformin effects on host metabolism and lifespan. Transgenic reporter strains (green) were used to quantify the expression of the following genes: *atgl-1*, required to mobilize fatty acids from triglyceride stores; *acs-2*, required for fatty acid activation; *cpt-5* and *cpt-2*, required for transport of fatty acids across the mitochondrial membrane; *acad-10*, a mitochondrial β−oxidation enzyme and *dhs-23*, a peroxisomal short chain dehydrogenase involved in steroid and lipid metabolism. Genetic mutants or RNAi knockdown (orange) were used to investigate the role of the following genes: *nhr-49*, a global regulator of β−oxidation; *acs-1*, a mitochondrial β−oxidation enzyme; *acox-1.1*/*5*, peroxisomal β−oxidation enzymes; *fzo-1* and *eat-3*, required for mitochondrial fusion; *drp-1*, required for mitochondrial fission; *nuo-1*, *gas-1*, *isp-1* and *cco-1*, required for electron transport chain function and *prx-5*, required for peroxisomal biogenesis. Lipid droplets and peroxisomes were visualized using transgenic strains that report the *dhs-3* lipid droplet marker protein and a RFP-PTS1 peroxisome-targeting sequence fusion, respectively (blue). Worms were also treated with perhexiline, an inhibitor of β−oxidation and acetoacetate, a product of fatty acid β−oxidation. (E and F) Confocal visualization of worm lipid droplets (E) and peroxisomes (F) show effects of metformin in worms in a bacterial OP50-MR-dependent manner. 10 μm scale bar. No changes in gene expression for *dhs-3* or *vha-6* were observed (Table S6). (G) Metformin increases worm peroxisomal abundance in a bacterial OP50-MR-dependent manner. Significance stars represent metformin effect (purple) and metformin-bacteria interaction (green). Data are represented as mean ± SEM n.s.- non-significant, ^∗^p < 0.05; ^∗∗^p < 0.01; ^∗∗∗^p < 0.001. See also Table S1 for lifespan statistics and Table S6 for RNA-seq statistics.

**Figure 7:**
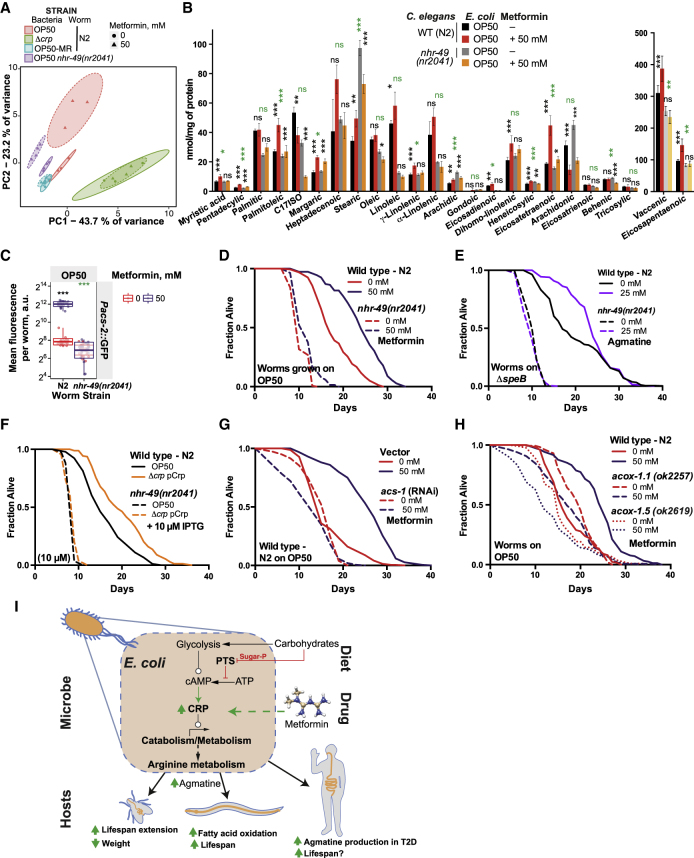
Metformin Increases Fatty Acid Oxidation to Regulate Host Metabolism and Lifespan (A) PCA plot of fatty acid metabolomics data, showing distinct signatures of metformin in worms in a bacterium- and worm *nhr-49*-dependent manner. (B) Fatty acid metabolomics in worms, showing an interaction between metformin and worm *nhr-49*. (C–F) Host *nhr-49* regulates metformin effects on worm *Pacs-2*::GFP expression (C) and the effects of metformin (D), agmatine supplementation (E), and *E. coli* OP50 Crp overexpression (F) on the worm lifespan. (G and H) Worm lifespan extension by metformin is abolished by RNAi knockdown of the mitochondrial FAO gene *acs-1* (G) and in *acox-1.1* and *acox-1.5* peroxisomal FAO mutants (H). (I) Proposed model of host-microbe-drug-nutrient interactions that regulate metformin effects on host metabolism and lifespan. Data are represented as mean ± SEM. ^∗^p < 0.05, ^∗∗^p < 0.01, ^∗∗∗^p < 0.001. In (B) and (C), significance stars represent metformin effect (black) and metformin-genotype interaction (green). See also Table S1 for lifespan statistics and Table S7 for fatty acid metabolomics statistics.

**Figure S7:**
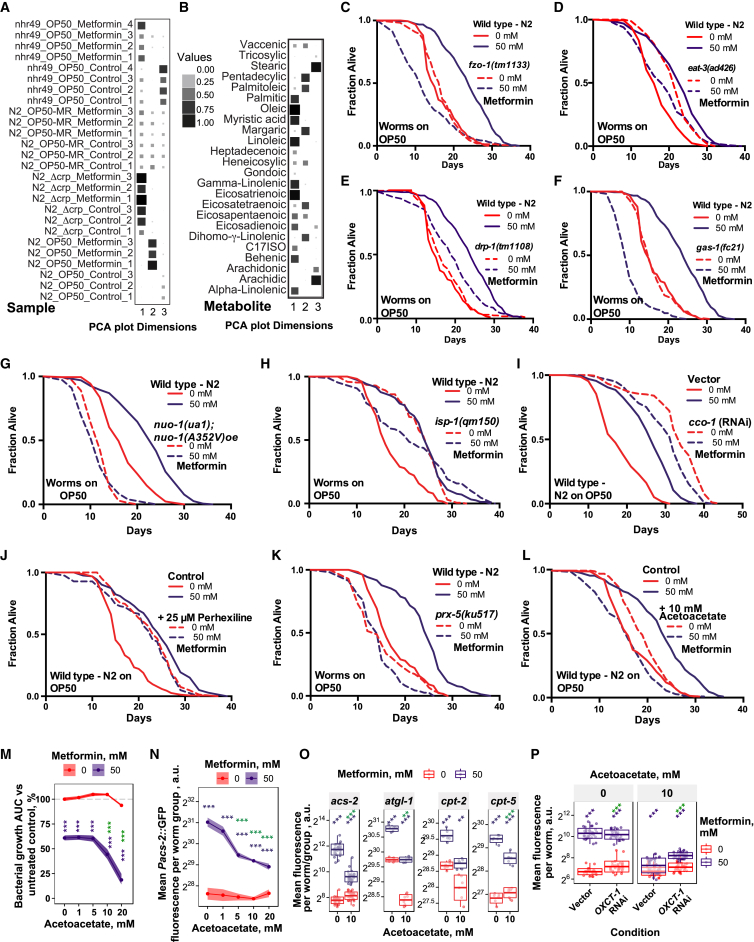
Metformin Increases Fatty Acid Oxidation to Regulate Host Metabolism and Lifespan, Related to Figure 7 (A and B) Quality of representation (measured as squared cosine) of the variables (Samples in (A) and metabolites in (B)) in the first three Principle Components. Value ranges between 0 and 1, where 1 corresponds to the maximum quality of representation. (C–E) Worm lifespan extension by metformin is suppressed in *fzo-1* (C) and *eat-3* (D) mitochondrial fusion mutants and a *drp-1* mitochondrial fission mutant (E) involved in mitochondrial homeostasis. (F–I) Worm lifespan extension by metformin is suppressed in *gas-1* (F) and *nuo-1* (G) mitochondrial respiration complex I mutants, an *isp-1* mitochondrial complex III mutant (H) and with RNAi knockdown of *cco-1* encoding a mitochondrial complex IV subunit (I). (J) Metformin does not further extend lifespan of worms treated with the FAO-inhibitor perhexiline (control plates supplemented with 0.25% DMSO). (K) Worm lifespan extension by metformin is suppressed in a *prx-5* peroxisomal biogenesis mutant. (L) Worm lifespan extension by metformin is abolished by acetoacetate supplementation. (M) Acetoacetate synergizes with metformin to inhibit *E. coli* OP50 growth. Significance stars represent metformin effect (purple) and metformin-acetoacetate interaction (green). (N) Acetoacetate supplementation suppresses metformin-induced upregulation of worm *Pacs-2*::GFP expression in a concentration-dependent manner. Significance stars represent metformin effect (purple) and metformin-acetoacetate interaction (green). (O) Acetoacetate supplementation suppresses metformin-induced upregulation of multiple worm lipid metabolism and FAO-related genes. Significance stars represent metformin effect (purple) and metformin-acetoacetate interaction (green). (P) Suppression of metformin-induced upregulation of worm *Pacs-2*::GFP expression by acetoacetate is partially rescued by RNAi knockdown of Succinyl-CoA:3-Ketoacid-CoA Transferase OXCT-1/C05C10.3, a gene involved in the catabolism of ketone bodies including acetoacetate. This suggests that effect of acetoacetate partly depends on its utilization as metabolic fuel. Significance stars represent metformin effect (purple) and metformin-OXCT-1 interaction (green). Data are represented as mean ± SEM. ^∗∗^p < 0.01; ^∗∗∗^p < 0.001. See also Table S1 for lifespan statistics and Table S7 for fatty acid metabolomics statistics.

To identify the molecular processes and genes that mediate the Crp and agmatine-dependent effects of metformin on organismal longevity, we performed a multi-omic analysis on the *C. elegans* host. RNA sequencing (RNA-seq) analyses (Figures 6A, 6B, and S6A), validated using transcriptional reporter lines (Figures S6B and S6C), showed that metformin treatment induced distinct worm transcriptional profiles in a bacterial strain-dependent manner. Among the most significantly enriched KEGG terms associated with the genes responsible for the metformin-induced longevity phenotype were processes involving peroxisomal (FDR = 2.7 × 10^−4^) and fatty acid metabolism (FDR = 1.5 × 10^−8^) (Figure 6B; Table S6). To assess transcriptional and cellular changes *in vivo* induced by metformin and bacteria, we used worm transgenic reporter lines for genes associated with lipid metabolism that mark lipid droplets and peroxisomes in the intestine (Figure S6D). Metformin upregulated genes involved in lipid metabolism (Figures 6C, S6B, and S6C) and specifically decreased lipid droplet size and abundance (Figures 6D and S6E) while increasing peroxisomal abundance (Figures 6E, S6F, and S6G) in worms grown on OP50. Significantly, this effect was not seen in worms grown on Δ*crp* bacteria. We also performed a metabolomic analysis of free and bound fatty acids and found that metformin significantly altered 16 of the 24 fatty acids measured (Figure 6F; Table S7). Changes in *C. elegans* lipid profiles induced by metformin were abolished in worms grown on Δ*crp* bacteria (Figures 7A, S7A, and S7B). These observations are consistent with a key role of bacterial Crp in mediating the effects of metformin on host lipid metabolism.

We hypothesized that bacterium-dependent changes in host fatty acid oxidation (FAO) metabolism are causally linked to metformin-induced longevity. Therefore, we investigated the role of the global fatty acid oxidation transcriptional regulator, the host nuclear hormone receptor NHR-49/PPARα ortholog (Figures 7C–7F), as well as genes directly involved in mitochondrial and peroxisomal fatty acid oxidation metabolism (Figures 7G and 7H) and in processes such as mitochondrial respiration and homeostasis (Figures S7C–S7I) that also regulatefatty acid oxidation (Figure S6C; Weir et al., 2017). In support of our hypothesis, a mutation in the worm gene *nhr-49* suppressed the fatty acid metabolic signature induced by metformin (Figures 7A, 7B, S7A, and S7B; Table S7) and upregulation of the fatty acid oxidation gene *acs-2* by metformin (Figure 7C). The *nhr-49* mutation also abolished the lifespan extension induced by metformin (Figure 7D), agmatine supplementation (Figure 7E), and bacterial CRP overexpression (Figure 7F). The lifespan-extending effects of metformin on worms were also abolished by RNAi knockdown of the mitochondrial fatty acid oxidation enzyme *acs-1*/ACSF2 (Figure 7G), pharmacological inhibition of mitochondrial fatty acid oxidation by perhexiline (Figure S7J; Kim et al., 2016), genetic deletion of the peroxisomal fatty acid oxidation enzymes *acox-1.1* /ACOX1 or *acox-1.5*/ACOX1 (Figure 7H), genetic deletion of the peroxisomal biogenesis gene *prx-5* (Figure S7K), and supplementation with the fatty acid oxidation product acetoacetate (Puchalska and Crawford, 2017; Figures 1E, S1I, S1J, and S7L–S7P).

Altogether, our data highlight a new role for bacterial signaling via Crp and bacterium-derived metabolites, including agmatine, in mediating links between metformin treatment and host lipid metabolism to regulate longevity (Figure 7I).

## Discussion

Moving away from correlative descriptions to in-depth mechanistic studies that establish a causative role for microbiota and their metabolites on host physiology is a highly desirable aim of both fundamental and applied research with wider implications for personalized medicine (Fischbach, 2018, Schmidt et al., 2018). We used a high-throughput four-way host-microbe-drug-nutrient screen and an *in silico* human microbiota metabolic modeling approach to identify bacterial effectors of metformin response in the host. We find that bacteria integrate nutrient and drug cues via a metabolic signaling axis involving the PTS, required for sugar uptake, and the downstream transcription factor Crp. A detailed mechanistic investigation points to agmatine as a bacterially produced metabolite required for the effects of metformin on host lipid metabolism and lifespan in both *C. elegans* and *D. melanogaster* (Figure 7I). Thus, our study dissects the evolutionarily conserved links between microbe-derived metabolites and the host and their modulation by environmental cues such as drugs and nutrition.

The host, its microbes, and the environment form a single physiological unit of study, with all of its parts being either targets of drugs or potential regulators of drug action. Therefore, understanding the complex interactions between host genetics, microbial genetics, and the environment (e.g., drugs and nutrition) requires a holistic approach to pharmacology and a paradigm shift toward holobiont-focused research (Kundu et al., 2017). Within this framework, our four-way screening approach investigating host-microbe-drug-nutrient interactions on an extensive scale provides an experimental tool for achieving such a holistic understanding. It revealed an unexpected mechanism of bacterial metabolic adaptation to metformin involving the signaling axis PTS-Crp, which indirectly regulates the host lifespan and is modulated by the nutritional landscape (Figure 7I). Analogous to the effects of metformin on eukaryotic cells, where activation of the metabolic rheostat AMP-activated protein kinase (AMPK) by metformin shifts cellular metabolism to a catabolic state (Pryor and Cabreiro, 2015), metformin-mediated enhancement of PTS-Crp signaling in prokaryotic cells (Chubukov et al., 2014) increased bacterial catabolism and indirectly increased the lifespan in both *C. elegans* and *Drosophila*. Furthermore, the powerful combination of our experimental systems with *in silico* metabolic modeling approaches allowed prediction of microbiome species involved in the production of metabolites that regulate host physiology (e.g., *E. coli* production of the polyamine precursor agmatine in a nutrient-dependent manner). In support of this functional role of the microbiota in the context of metformin, our cohort-based microbial community modeling study, including patient-specific dietary information, showed that metformin treatment was significantly associated with a predicted increase in agmatine production capacity by *E. coli* and other Enterobacteriales across cohorts as well as longitudinally. Our findings are consistent with previous studies showing that metformin partly exerts its therapeutic effects by selectively altering the profile of the human gut microbiota to improve dysbiosis associated with type 2 diabetes, in particular by increasing the abundance of *Escherichia* (Forslund et al., 2015, Wu et al., 2017). The current results point to a key role of agmatine in mediating the effects of metformin. However, because it is currently not possible to measure agmatine production by microbes in either model organisms or humans, future work is need to directly link agmatine production with increased longevity of metformin-treated type 2 diabetic humans compared with matched non-diabetic controls (Barzilai et al., 2016).

Altogether, our four-way screen approach combined with *in silico* microbial community modeling captures functional features in microbes induced by metformin that are conserved from worms to humans and provides an experimental strategy for future investigations into complex host-microbe-drug-nutrient interactions. Because the microbiota is an attractive target for therapeutic intervention (Ho et al., 2018), understanding how the nutritional environment regulates drug action through the microbiota and elucidating the underlying metabolic pathways through metabolic modeling may help inform dietary guidelines that promote maximum drug efficacy and reduce gastrointestinal side effects. Given the myriad of untapped drug-nutrient-microbe interactions, orchestrating bacterial metabolic responses through drug-signaling interactions may yield a promising avenue for personalized medicine aimed at improving host health and longevity.

## STAR★Methods

### Key Resources Table

**Table:** 

REAGENT or RESOURCE	SOURCE	IDENTIFIER
**Antibodies**		

Purified anti-*E. coli* Crp	BioLegend	Cat# 664304; RRID: AB_2565553
Purified anti-*E. coli* RNA Sigma 70	BioLegend	Cat# 663202; RRID: AB_2564410
Goat anti-mouse IgG	Sigma-Aldrich	Cat# A0168; RRID: AB_257867

**Bacterial and Virus Strains**		

*E. coli*: OP50	CGC	RRID:WB-STRAIN:OP50
*E. coli*: OP50-MR (metformin resistant)	Cabreiro et al., 2013	N/A
*E. coli:* OP50(xu363)	CGC	RRID:WB-STRAIN: OP50(xu363)
*E. coli:* OP50 *ΔptsHΔptsIΔCrr::kan*	Cynthia Kenyon (Lee et al., 2009)	N/A
Keio collection: Single-gene knockout mutants in *E. coli* BW25113 background	NBRP	https://shigen.nig.ac.jp/ecoli/strain/resource/keioCollection/list/
*E. coli:* OP50 *ΔadiA::kan*	This study	N/A
*E. coli:* OP50 *ΔarcA::kan*	This study	N/A
*E. coli:* OP50 *ΔargG::kan*	This study	N/A
*E. coli:* OP50 *ΔargR::kan*	This study	N/A
*E. coli:* OP50 *ΔastA::kan*	This study	N/A
*E. coli:* OP50 *ΔcpdA::kan*	This study	N/A
*E. coli:* OP50 *Δcra::kan*	This study	N/A
*E. coli:* OP50 *Δcrp::kan*	This study	N/A
*E. coli:* OP50 *Δcrr::kan*	This study	N/A
*E. coli:* OP50 *ΔcsiR::kan*	This study	N/A
*E. coli:* OP50 *ΔcyaA::kan*	This study	N/A
*E. coli:* OP50 *Δfur::kan*	This study	N/A
*E. coli:* OP50 *ΔgcvA::kan*	This study	N/A
*E. coli:* OP50 *ΔgcvH::kan*	This study	N/A
*E. coli:* OP50 *ΔgcvP::kan*	This study	N/A
*E. coli:* OP50 *ΔgcvT::kan*	This study	N/A
*E. coli:* OP50 *ΔglpK::kan*	This study	N/A
*E. coli:* OP50 *Δhpt::kan*	This study	N/A
*E. coli:* OP50 *ΔmarA::kan*	This study	N/A
*E. coli:* OP50 *Δmlc::kan*	This study	N/A
*E. coli:* OP50 *Δnac::kan*	This study	N/A
*E. coli:* OP50 *ΔntrC::kan*	This study	N/A
*E. coli:* OP50 *ΔrbsK::kan*	This study	N/A
*E. coli:* OP50 *ΔspeA::kan*	This study	N/A
*E. coli:* OP50 *ΔspeB::kan*	This study	N/A
*E. coli:* OP50 *ΔspeF::kan*	This study	N/A
*E. coli:* OP50 *ΔtdcB::kan*	This study	N/A
*E. coli:* OP50 *ΔtdcC::kan*	This study	N/A
*E. coli:* OP50 *ΔcpdAΔcrp::kan*	This study	N/A
*E. coli:* OP50 *ΔcpdAΔcyaA::kan*	This study	N/A
*E. coli:* OP50 *ΔspeAΔadiA::kan*	This study	N/A
*E. coli:* OP50 *ΔspeAΔspeB::kan*	This study	N/A
*E. coli:* OP50 *ΔspeBΔastA::kan*	This study	N/A
*E. coli*: OP50 Δ*adiA*Δ*astA*Δ*speA*::*kan*	This study	N/A
*E. coli:* OP50 Δ*adiAΔastAΔspeAΔspeB::kan*	This study	N/A
*E. coli:* OP50 pCrp	This study	N/A
*E. coli:* OP50 pAstC	This study	N/A
*E. coli:* OP50 pFepA	This study	N/A
*E. coli:* OP50 pFucI	This study	N/A
*E. coli:* OP50 pGalF	This study	N/A
*E. coli:* OP50 pGapA	This study	N/A
*E. coli:* OP50 pMdh	This study	N/A
*E. coli:* OP50 pMntR	This study	N/A
*E. coli:* OP50 pOmpA	This study	N/A
*E. coli:* OP50 pSodA	This study	N/A
*E. coli:* OP50 pUbiF	This study	N/A
*E. coli:* OP50 pYdcS	This study	N/A
*E. coli:* OP50 pYbjJ	This study	N/A
*E. coli*: OP50 Δ*crp*::*kan* pCrp	This study	N/A
*E. coli:* OP50 *ΔcyaA::kan* pCrp	This study	N/A

**Chemicals, Peptides, and Recombinant Proteins**		

5-Fluoro-2′-deoxyuridine 98+%	Alfa Aesar	Cat# L16497
Adenosine ≥ 99%	Sigma-Aldrich	Cat# A9251
Adenosine 3′,5′-cyclic monophosphate (cAMP)	Acros Organics	Cat# 225800010
Agmatine sulfate 97%	Alfa Aesar	Cat# H55363
Agar	Sigma-Aldrich	Cat# A7002
Bacto peptone	BD Biosciences	Cat# 211677
CHAPS	GE Healthcare	Cat# 17-1314-01
cOmplete protease inhibitor cocktail	Roche	Cat# 11697498001
D-(+)-Glucose ≥ 99.5%	Sigma-Aldrich	Cat# G8270
D-(−)-Ribose ≥ 99%	Sigma-Aldrich	Cat# R7500
Dithiothreitol (DTT)	GE Healthcare	Cat# 17-1318-01
Glycerol ≥ 99.5%	Thermo Fisher Scientific	Cat# BP229-1
Iodoacetamide (IAA)	Fluka	Cat# 57670
Isopropyl-β-D-thiogalactopyranoside (IPTG) ≥ 99%	Thermo Fisher Scientific	Cat# BP1755
L-Serine	Sigma-Aldrich	Cat# S4500
LB Broth Miller	Fisher BioReagents	Cat# BP1426
Lithium acetoacetate ≥ 90%	Sigma-Aldrich	Cat# A8509
Metformin (1,1-Dimethylbiguanide hydrochloride) ≥ 98%	LKT Laboratories	Cat# M2076
MRS broth	BD Difco	Cat# 288130
Perhexiline maleate salt ≥ 98%	Sigma-Aldrich	Cat# SML0120
Sequencing grade modified trypsin	Promega	Cat# V5111
Soy peptone	Sigma-Aldrich	Cat# P6713
Thiourea	Sigma-Aldrich	Cat# T8656
Tryptone	BD Biosciences	Cat# 211705
Urea	GE Healthcare	Cat# 17-1319-01
Yeast extract	BD Biosciences	Cat# 288620

**Critical Commercial Assays**		

Biolog Phenotype Microarrays PM1, PM2A, PM3B, PM4A	Biolog	Cat# 12191
Biolog Dye mix A	Biolog	Cat# 74221
Clarity Western ECL Substrate	Bio-Rad	Cat# 1705060
CyDye DIGE Fluor Minimal Labeling Kit	GE Healthcare	Cat# 25-8010-65
Direct-zol RNase Miniprep Kit	Zymo Research	Cat# R2060
GenElute Plasmid Miniprep Kit	Sigma-Aldrich	Cat# PLN70
PlusOne Mini Dialysis Kit, 1kDa	GE Healthcare	Cat# 80648394
Qubit Protein Assay Kit	Thermo Fisher Scientific	Cat# Q33211
Qubit RNA HS Assay Kit	Thermo Fisher Scientific	Cat# Q32852
Quick Start Bradford Protein Assay Kit	Bio-Rad	Cat# 5000201

**Deposited Data**		

C. elegans RNA-Seq reads and read counts.	This study	ArrayExpress: E-MTAB-7272
*E. coli* western blot images	This study	Mendeley Data https://data.mendeley.com/datasets/crmtpmd622/draft?a=ef347ccd-7532-44b0-8925-d2c04a71b419
*C. elegans* confocal microscopy images	This study	Mendeley Data https://data.mendeley.com/datasets/crmtpmd622/draft?a=ef347ccd-7532-44b0-8925-d2c04a71b419
*C. elegans* and *D. melanogaster* lifespan data	This study	Table S1
4-way screen data	This study	Table S2
*E. coli* proteomics data	This study	Table S3
*E. coli* metabolomics data	This study	Table S4
*E. coli* and human microbiota metabolic data	This study	Table S5
*C. elegans* RNA-seq data	This study	Table S6
*C. elegans* fatty acid metabolomics data	This study	Table S7
Kiel cohort data (16S rRNA gene sequencing)	This study	Available upon application from the PopGen biobank (https://www.uksh.de/p2n/Information+for+Researchers.html)
Spanish cohort data (metagenomics sequencing)	Wu et al., 2017	PRJNA361402
Swedish cohort data (metagenomics sequencing)	Forslund et al., 2015, Karlsson et al., 2013	PRJEB1786
Danish cohort data (metagenomics sequencing)	Le Chatelier et al., 2013	PRJEB5224 PRJEB1220 PRJEB4336 PRJEB2054
Mapped microbial abundances for metagenome-based cohort data	This study	Mendeley Data; https://data.mendeley.com/datasets/crmtpmd622/draft?a=ef347ccd-7532-44b0-8925-d2c04a71b419
Refined AGORA models with agmatine transporters and extracellular agmatine production	This study	Mendeley Data; https://data.mendeley.com/datasets/crmtpmd622/draft?a=ef347ccd-7532-44b0-8925-d2c04a71b419
*E. coli* OP50 metabolic model	Zimmermann et al., 2019	N/A

**Experimental Models: Organisms/Strains**		

*C. elegans*: N2 Bristol	CGC	CGC: 10570
*C. elegans*: STE68: *nhr-49(nr2041)* I	CGC	RRID:WB-STRAIN:STE68
*C. elegans*: BC11281: *dpy-5(e907)* I sEx11281[*rCes* R07H5.2::GFP + *pCeh361*]	CGC	RRID:WB-STRAIN:BC11281
*C. elegans*: BX113: *lin-15*B&*lin-15*A*(n765)* X, waEx15[*Pfat-7*::GFP + *lin15*(+)]	CGC	RRID:WB-STRAIN:BX113
*C. elegans*: BC12124: sEx12124[R08H2.1::GFP]	CGC	RRID:WB-STRAIN:BC12124
*C. elegans*: VS10: hjIs37[*Pvha-6*::mRFP-PTS1 + *Cbr- unc-119(+)*]	CGC	RRID:WB-STRAIN:VS10
*C. elegans*: LIU1: ldrIs1[*Pdhs-3*::*dhs-3*::GFP + *unc-76*(+)]	CGC	RRID:WB-STRAIN:LIU1
*C. elegans*: CW152: *gas-1(fc21)* X	CGC	RRID:WB-STRAIN: CW152
*C. elegans*: CU5991: *fzo-1(tm1133)* II	CGC	RRID:WB-STRAIN: CU5991
*C. elegans*: VC1785: F08A8.1*(ok2257)* I	CGC	RRID:WB-STRAIN: VC1785
*C. elegans*: RB1985: C48B4.1*(ok2619)* III	CGC	RRID:WB-STRAIN: RB1985
*C. elegans*: MH5239: *prx-5(ku517)* II	CGC	RRID:WB-STRAIN: MH5239
*C. elegans*: CU6372: *drp-1(tm1108)* IV	CGC	RRID:WB-STRAIN: CU6372
*C. elegans*: CU5991: *fzo-1(tm1133)* II,	CGC	RRID:WB-STRAIN: CU5991
*C.* elegans: DA631: *eat-3(ad426)* II*; him-8(e1489)* IV	CGC	RRID:WB-STRAIN: DA631
*C. elegans*: MQ887: *isp-1(qm150)* IV	CGC	RRID:WB-STRAIN: MQ887
*C. elegans*: MAH547: sqEx82[*Pargk-1*::GFP+*rol-6(su1006)*]	CGC	RRID:WB-STRAIN:MAH547
*C. elegans*: LB54: *nuo-1(ua-1)* II, *unc-119(ed3)* III, *uaEx25*[p016bA352V], *uaEx32*[pDP#SU006, pTG96, pPD118.25NEO]	Bernard Lemire (DeCorby et al., 2007)	N/A
*C. elegans*: WBM392: wbmIs33[*Pacs-2*::GFP+*rol-6(su1006)*]	William Mair	N/A
*C. elegans*: SSR896: ssrIs496[P*atgl1*::GFP+*rol-6(su1006)*]	Supriya Srinivasan (Hussey et al., 2017)	N/A
*C. elegans*: MGH249: alxIs19[*PCeACAD10*::*CeACAD10*::mRFP3-HA *Pmyo-2*::GFP] 8X	Alexander Soukas (Wu et al., 2016)	N/A
*C. elegans*: GA641: wuIs177[*Pftn-1*::GFP+*lin-15*(+)]	David Gems (Ackerman and Gems, 2012)	N/A
*C. elegans*: FGC59: *nhr-49(nr2041)* I, wbmEx57[*Pacs-2*::GFP+*rol-6(su1006)*]	This study (Burkewitz et al., 2015)	N/A
*C. elegans*: FGC54: fgcIs1[*Pcpt-5*:: GFP+*rol-6(su1006)*]	This study	N/A
*C. elegans*: FGC45: ijIs10[*unc-76*(+)+*Pcpr-5*::GFP::*lacZ*]	This study and CGC	RRID:WB-STRAIN: IA123
*C. elegans*: FGC42: nIs470(*Pcysl-2*::GFP+*Pmyo-2*::mCherry]	This study and Robert Horvitz (Ma et al., 2012)	N/A
*Drosophila melanogaster*: *white Dahomey* (*w*^*Dah*^) WT	Linda Partridge	N/A

**Oligonucleotides**		

For information regarding oligonucleotide sequences used in this study please refer to Table S8.	This Study	Table S8

**Recombinant DNA**		

Ahringer *C. elegans* RNAi library: RNAi control plasmid: pL4440	Source BioScience	http://www.sourcebioscience.com/products/life-science-research/clones/rnai-resources/c-elegans-rnai-collection-ahringer/
Ahringer *C. elegans* RNAi library: RNAi *acs-1* knockdown: pL4440-*acs-1*	Source BioScience	http://www.sourcebioscience.com/products/life-science-research/clones/rnai-resources/c-elegans-rnai-collection-ahringer/
Ahringer *C. elegans* RNAi library: RNAi *cco-1* knockdown: pL4440-*cco-1*	Source BioScience	http://www.sourcebioscience.com/products/life-science-research/clones/rnai-resources/c-elegans-rnai-collection-ahringer/
Vidal *C. elegans* RNAi library: RNAi OXCT-1/C05C10.3 knockdown: pL4440- OXCT-1/C05C10.3	Source BioScience	http://www.sourcebioscience.com/products/life-science-research/clones/rnai-resources/c-elegans-orf-rnai-resource-vidal/
ASKA collection *E. coli* ORF clones (GFP -): Crp overexpression: pCrp	NBRP	https://shigen.nig.ac.jp/ecoli/strain/resource/askaClone/list/ASKA_CLONE_MINUS
pCP20	CGSC	CGSC: 7629
pKD4	Datsenko and Wanner, 2000	Addgene Cat# 45605
pKD46	CGSC	CGSC: 7739
pPD95.75	Hilbert and Kim, 2018	Addgene Cat# 1494

**Software and Algorithms**		

HISAT2 (v2.05)	Kim et al., 2015	https://ccb.jhu.edu/software/hisat2/index.shtml
R (v3.5.0)	R Core Team	https://www.r-project.org
edgeR (v3.22.0)	Robinson et al., 2010	https://bioconductor.org/packages/release/bioc/html/edgeR.html
DeCyder 7.0	GE Healthcare	Cat# 11505804
DESeq2	Love et al., 2014	https://bioconductor.org/packages/release/bioc/html/DESeq2.html
lme4 (v1.1-19)	https://cran.r-project.org/web/packages/lme4/index.html	https://cran.r-project.org/web/packages/lme4/index.html
Sybil (v2.0.4),	Gelius-Dietrich et al., 2013	https://cran.r-project.org/web/packages/sybil/index.html
cutadapt (v1.12)	10.14806/ej.17.1.200	https://cutadapt.readthedocs.io/en/stable/
prinseq lite (v0.20.4)	Schmieder and Edwards, 2011	http://prinseq.sourceforge.net/
Samtools (v1.4)	Li et al., 2009	http://www.htslib.org/
Peaks 7.5	Bioinformatics Solutions	http://www.bioinfor.com/peaks-studio-7-5-release/
Python (v2.7.13)	Python Core Team	https://www.python.org
GraphPad Prism 6	GraphPad Software	https://www.graphpad.com/scientific-software/prism/
JMP 12	SAS Institute	https://www.jmp.com/en_be/software/data-analysis-software.html

### Lead Contact and Materials Availability

All *E. coli* strains generated in this study will be made available upon request to the Lead Contact. The *C. elegans* strains FGC42, FGC45, FGC54 and FGC59 generated in this study will be made available upon request to the Lead Contact. Further information and requests for resources may be directed to, and will be fulfilled by the Lead Contact, Filipe Cabreiro (f.cabreiro@lms.mrc.ac.uk).

### Experimental Model and Subject Details

#### Nematode, Bacterial and Fly Strains

The following *C. elegans* strains were obtained from the CGC: N2 Bristol (wild-type), STE68 *nhr-49(nr2041)* I, BC11281 *dpy-5(e907)* I sEx11281[rCes R07H5.2::GFP + pCeh361], BX113 *lin-15*B&*lin-15*A*(n765)* X, waEx15[*Pfat-7*::GFP + *lin15*(+)], BC12124 sEx12124[R08H2.1::GFP], VS10 hjIs37[*Pvha-6*::mRFP-PTS1 + Cbr- *unc-119*(+)], LIU1 ldrIs1[*Pdhs-3*::*dhs-3*::GFP + *unc-76*(+)], CW152 *gas-1(fc21)* X, CU5991 *fzo-1(tm1133)* II, VC1785 F08A8.1*(ok2257)* I, RB1985 C48B4.1*(ok2619)* III, MH5239 *prx-5(ku517)* II, CU6372 *drp-1(tm1108)* IV, CU5991 *fzo-1(tm1133)* II, DA631 *eat-3(ad426)* II; *him-8(e1489)* IV, MQ887 *isp-1(qm150)* IV and MAH547 sqEx82[*Pargk-1*::GFP+*rol-6(su1006)*]. The LB54 *nuo-1(ua1)* II, *unc-119(ed3)* III, *uaEx25*[p016bA352V], *uaEx32*[pDP#SU006, pTG96, pPD118.25NEO] strain, which was a gift from Bernard Lemire, is homozygous for the lethal *nuo-1(ua1)* allele and carries an extrachromosomal array with a Ala352Val substituted *nuo-1* gene. This point mutation has been shown to reduce complex I activity to approximately 30% of WT (DeCorby et al., 2007). The WBM392 wbmIs33[*Pacs-2*::GFP+*rol-6(su1006)*] strain was a gift from William Mair. The SSR896 ssrIs496[P*atgl-1*::GFP+*rol-6(su1006)*] strain was a gift from Supriya Srinivasan. The MGH249 alxIs19[*PCeACAD10*::*CeACAD10*::mRFP3-HA +*Pmyo-2*::GFP] 8X strain was a gift from Alexander Soukas. The GA641 wuIs177[*Pftn-1*::GFP+*lin-15*(+)] strain was a gift from David Gems. The following strains were generated in this study: FGC59 *nhr-49(nr2041)* I, wbmEx57[*Pacs-2*::GFP+*rol-6(su1006)*], FGC54 fgcEx1[*Pcpt-5*::GFP+*rol-6(su1006)*], FGC45 ijIs10[*unc-76*(+)+*Pcpr-5*::GFP::lacZ] and FGC42 nIs470(*Pcysl-2*::GFP+*Pmyo-2*::mCherry] from outcrossing the strain MT20664, a gift from Robert Horvitz.

*E. coli* strains used in this study include OP50, obtained from the CGC, and OP50-MR (Cabreiro et al., 2013). The OP50 Δ*ptsHIcrr* strain was a gift from Cynthia Kenyon. OP50 deletion mutants were created using the *E. coli* Keio Knockout Collection (odd numbered strains), obtained from the National BioResource Project. OP50 overexpressor strains were created using the *E. coli* ORF ASKA collection, also obtained from the National BioResource Project. RNAi knockdown was performed using OP50(xu363) transformed with the *acs-1* and *cco-1* RNAi plasmids obtained from the Ahringer library, the OXCT-1/C05C10.3 RNAi plasmid obtained from the Vidal library and the L4440 empty vector control. Details of all *E. coli* strains generated in this study can be found in the Key Resources Table.

The *Drosophila melanogaster white Dahomey* (*w*^*Dah*^) wild-type strain used in this study was collected in 1970 in Dahomey (now Benin) and has since been maintained in large population cages with overlapping generations.

#### Nematode Culture Conditions

Worms were maintained at 20°C, unless otherwise stated, on nematode growth medium (NGM) seeded with *E. coli*. Where indicated, molten agar was supplemented with the following compounds: metformin (6.25, 12.5, 25, 50, 100 mM), acetoacetate (1, 5, 10, 20 mM), D-ribose (0.2%), glycerol (0.2%), L-serine (50 mM), adenosine (2 mM), glucose (0.2%), IPTG (10, 25, 50 μM), cAMP (1 mM) and agmatine (10, 25, 50 mM). Where indicated, the composition of NGM was modified so that Bacto peptone was replaced with the equivalent mass of either soy peptone, LB (2:1 tryptone and yeast extract) or MRS medium. For perhexiline treatment, a 100 mM stock solution was made in 100% DMSO and then diluted to 2.5 mM in water. 100 μL of 2.5 mM perhexiline was added topically to bacterial lawns (final concentration 25 μl) 1 hour before transferring worms. Similarly, 100 μL of 2.5% DMSO was added to control plates. For maintenance of the LB54 *nuo-1(0)* mutant strain, worms were grown on plates supplemented with 1 mg/ml G418 (Geneticin) antibiotic to select for the retention of the extrachromosomal array. The antibiotic was not added to experimental plates used for lifespan analysis. For RNAi knockdown, RNAi bacterial strains were cultured overnight in LB supplemented with 100 μg/ml ampicillin and were seeded onto NGM plates supplemented with 1 mM IPTG to induce dsRNA expression.

#### Bacterial Culture Conditions

Bacterial strains were cultured by inoculating a single colony grown on LB agar in LB broth and incubated at 37°C overnight (approximately 16 hours). Where appropriate, LB was supplemented with 50 μg/ml kanamycin, 30 μg/ml chloramphenicol or 100 μg/ml ampicillin. Kanamycin and chloramphenicol were not added to liquid cultures if the bacteria was cultured for use with *C. elegans* to avoid possible detrimental effects associated with antibiotic exposure.

#### Nematode Strain Construction

The *Pcpt-5*::GFP reporter strain was generated using a construct made by PCR fusion. The promoter region of *cpt-5* was amplified from worm lysate using F: 5′- GTCTCGGAATTGATGCATAG-3′ and R: 5′- AGTCGACCTGCAGGCATGCAAGCTTTTTCACTGCAAATTTCAATCTAT-3′ primers. GFP was amplified from the pPD95.75 GFP expression vector using F: 5′- AGCTTGCATGCCTGCAGGTC-3′ and R: 5′-AGGGCCCGTACGGCCGACTA-3′ primers. The products from these two reactions were fused using F: 5′-CAGAATTGGAAGTCTTACAGC-3′ and R: 5′-GGAAACAGTTATGTTTGGTATATTG-3′ nested primers. The resulting *Pcpt-5*::GFP construct was microinjected into the gonad of adult N2 worms at 1 mg/ml with 100 mg/ml of a rol-6 co-injection marker to obtain strain FGC54.

#### Bacterial Strain Construction

Bacterial strains generated in this study are listed in the Key Resource Table. *E. coli* OP50 single gene deletion mutants were created using P1 *vir* phage-mediated transduction to transfer kanamycin resistant-tagged mutations from *E. coli* K12 mutant strains obtained from the Keio collection into OP50. To introduce additional mutations, it was necessary to remove the kanamycin resistant marker by transformation with pCP20 before performing the transduction. Due to the close proximity of the *speA* and *speB* genes, it was necessary to create the Δ*speA*Δ*speB* double mutant using targeted gene disruption by homologous recombination. The kanamycin resistance gene was amplified from the pKD4 plasmid using F: 5′- ACGACATGTCTATGGGTTTGCCTTCGTCAGCGGGCGAATGTGTAGGCTGGAGCTG-3′ and R: 5′- TCGCCCTTTTTCGCCGCCTGAATATACAGCATTTCCAGCGCCATATGAATATCCTCCTTAGT-3′ primers. OP50 carrying the pKD46 plasmid expressing λ Red recombinase was transformed with the resulting DNA fragment. All bacterial gene deletion mutants were confirmed by colony PCR using the primers detailed in table S8. Primers were designed to bind upstream (-cseq-F) or downstream (-cseq-R) of the mutation site. Reactions were carried out using the –cseq-F primer in conjunction with the K1 reverse primer which binds to the kanamycin resistance gene. Alternatively, the – cseq-R primer could be used in conjunction with the K2 forward primer which also bind to the kanamycin resistance gene. Kanamycin-sensitive deletion mutants were confirmed by using the appropriate forward and reverse primers for the gene of interest and comparing the size of the fragment with that obtained for the wild-type control strain.

*E. coli* OP50 strains overexpressing Crp were created by transformation with a pCA24N plasmid that expresses Crp under the control of an IPTG-inducible promoter. This plasmid was extracted from an ASKA collection clone using a GenElute Plasmid MiniPrep Kit (Sigma-Aldrich). Successful transformation was confirmed by colony PCR. The -cseq-R primer was designed to bind internally to the crp gene and was used in conjunction with the ASKA-cseq-F primer that binds to the pCA24N plasmid.

*E. coli* strains used for RNAi knockdown were created by transforming OP50(xu363) with RNAi plasmids extracted from either the Ahringer or Vidal libraries using a GenElute Plasmid MiniPrep Kit (Sigma-Aldrich). The identity of RNAi plasmids was confirmed by sequencing using primers detailed in Table S8.

#### Human cohorts

Phenotypic details for human cohorts are provided in Table S5E.

##### Kiel cohort

We used samples from the Kiel-based cohorts PopGen (Krawczak et al., 2006) and FoCus (Müller et al., 2015). Information on medication and food supplement usage was recorded. Data and specimens from both cohorts were handled by the same biobank using a single study protocol. Access to the cohort data along with phenotypic information was granted by the PopGen biobank (Krawczak et al., 2006). For analysis, samples were grouped into four phenotypic groups: a) lean (BMI ≤ 25) without diabetes, IBD, or IBS, with fasting glucose level below 125 mg/dl (“Lean Healthy,” LH); b) obese (BMI > 30) with same criteria as LH except for BMI (“Obese Healthy,” ObH); c) obese (BMI > 30) with diagnosed T2D or fasting glucose level above 125 mg/dl without antidiabetic treatment, and without IBD and IBS, respectively (T2D Met-) and d) obese (BMI > 30) with diagnosed T2D or fasting glucose level above 125 mg/dl taking metformin, and without IBD and IBS, respectively (T2D Met+). Since type 2 diabetic patients not treated with metformin were significantly older than participants of the other groups, we iteratively removed the oldest sample from the untreated type 2 diabetic group until the median age of the remaining cohort matched the median age of the metformin-treated cohort (8 individuals removed). Samples and data for the Kiel cohort were provided by the PopGen Biobank (Schleswig-Holstein, Germany) and can be accessed via a structured application procedure (https://www.uksh.de/p2n/Information+for+Researchers.html).

##### Longitudinal Spanish cohort

Type 2 diabetes (T2D) patients were recruited at the Hospital Universitari Dr. Josep Trueta (Girona, Spain) and fecal genomic DNA sampled. The study was performed in a longitudinal design, where participants received either a placebo or metformin treatment. Sampling time points were at study entry and at month 2 and month 4. A subset of the participants within the placebo group were switched to metformin treatment after the end of the first study phase. Those participants were additionally followed up 6 months post starting metformin intake. The metagenomic data was originally published in Wu et al. (2017) and can be accessed via PRJNA361402.

##### Swedish cohort

The data was originally published in Karlsson et al. (2013) and reevaluated in Forslund et al. (2015). The data is available via the accession PRJEB1786. The Swedish study is a female-only cohort. The original study included normal and impaired glucose tolerant participants as part of the non-diabetic group. We only considered normal glucose tolerant participants for the healthy group.

##### Danish cohort

The Danish cohort consists of four independently published metagenomic datasets of the MetaHIT-project (Le Chatelier et al., 2013) also referred to as MHD-cohort in Forslund et al. (2015). In the first study 277 nondiabetic danish individuals were sampled for their gut microbiome followed by 75 T2D and 31 type 1 diabetic patients. All samples were sequenced using identical protocols. The data can be accessed from the European Nucleotide Archive with the project-IDs PRJEB5224 for type 1 and type 2 diabetic patients and PRJEB1220, PRJEB4336, PRJEB2054 for all non-disease controls. Data from type 1 diabetic patients was not considered.

### Method Details

#### Bacterial Growth Assay

Bacterial growth assays were performed in transparent, flat-bottomed 96-well plates. Unless otherwise stated, plates were prepared by loading each well with 200 μL of LB solution containing an overnight bacterial culture diluted 1000-fold and metformin/supplements at the desired concentration as required. If metformin or other supplements were added, an equivalent volume of water was added to negative control wells. To investigate the effect of different types of media on bacterial growth, LB was replaced with either standard liquid NGM made with Bacto peptone or modified liquid NGM containing either a soy peptone, LB or MRS base. To investigate the effect of glycerol and D-ribose supplementation on bacterial growth, cells harvested from overnight cultures were washed with liquid NGM prior to dilution and the assay was performed with standard liquid NGM. To investigate the effect of increasing IPTG concentrations on the growth of bacterial strains overexpressing Crp, the assay was performed with standard liquid NGM. The absorbance of each well was measured at OD 595 nm using a Tecan Infinite M2000 microplate reader operated via Magellan V6.5 software (Tecan). Measurements were taken every 5 minutes over an 18-hour period. Throughout this time, the plate was maintained at 37°C with constant shaking. At least 3 independent trials were carried out per experiment. Data was analyzed using R (R Core Team). The total bacterial growth was estimated as the OD area under the curve (AUC) integral. AUC values were *log*_*2*_ transformed to enable relative comparisons in logFC scale to be made. Statistical significance was assessed by either one-way or two-way ANOVA depending on the experimental design.

#### Western Blotting

Experimental NGM plates were seeded with 150 μL of overnight bacterial culture and lawns were left to grow at 20°C for 4 days for all experiments with the exception of those involving Crp overexpressor strains where lawns were left for 4 hours. Bacteria was collected into 2 mL microcentrifuge tubes using a cell scraper and was frozen in liquid nitrogen, then stored at −80°C. Cells were thawed and resuspended in 150 μL of buffer composed of B-PER reagent (Thermo Fisher Scientific) containing 40 mM dithiothreitol and 1X protease inhibitor cocktail (Roche). Cells were lysed using a Q700 sonicator waterbath (Qsonica) kept at 4°C with 5x15 s pulses at 100% amplitude. Lysates were centrifuged at maximum speed for 30 minutes at 4°C to pellet cellular debris and the resulting supernatant was transferred to fresh tubes. The protein concentration of each sample was determined using the Bradford assay. 40 μg of protein diluted in 10 μL of sample buffer was added to 10 μL of 2X laemmli buffer (Bio-Rad). Samples were heated at 95°C for 5 minutes and were loaded into a 4%–20% Criterion TGX precast gel (Bio-Rad) for SDS-PAGE. Separated proteins were transferred onto a nitrocellulose membrane. The membrane was probed with a purified anti-*E. coli* Crp primary antibody (BioLegend) at a 1:2000 dilution and an HRP conjugated goat anti-mouse IgG secondary antibody (Sigma-Aldrich) at a 1:5000 dilution. The membrane was exposed on film using Clarity Western ECL Substrate (Bio-Rad). The membrane was stripped by immersing in PLUS Western Blot Stripping Buffer (Thermo Fisher Scientific) for 15 minutes and was reprobed with an anti-*E. coli* RNA Sigma 70 Antibody (BioLegend) at a 1:2500 dilution to provide a loading control. Probing with secondary antibody and exposure of the membrane was carried out as before. Films were scanned and densitometry was performed using ImageJ software (NIH). Bands were detected manually and the background was subtracted from each peak generated. The intensity of each Crp band was normalized by the intensity of its corresponding sigma 70 band. 3-6 independent biological replicates were included per condition. Statistical analysis was performed by two-way ANOVA using GraphPad Prism 6 software.

#### Bacterial Proteomics

Control and 50 mM metformin-supplemented NGM plates were seeded with 150 μL of overnight bacterial culture and lawns were left to grow at 20°C for 4 days. 5-6 independent biological replicates were included per condition. Bacteria was collected from plates in S-basal medium using a sterile glass scraper. Samples were centrifuged at 12,000 g for 4 minutes at 4°C. The supernatant was removed and pellets were transferred to protein LoBind tubes (Eppendorf). 900 μL of chilled protein lysis buffer (7 M urea, 2 M thiourea, 4% CHAPS, 40 mM Tris supplemented with Roche cOmplete Protease Inhibitor) was added to the pellet. Samples were kept on ice from this point onward. Pellets were lysed via sonication for 2 × 10 s and proteins were separated from the cellular debris by centrifuging at 15,000 g for 12 minutes at 4°C. Supernatant containing the extracted protein was transferred to clean LoBind tubes and samples were desalted overnight using the PlusOne Mini Dialysis Kit (GE Healthcare). The protein concentration in each sample was determined using the Qubit Protein Assay (Invitrogen), and samples were aliquoted and stored at −80°C until further use.

Proteins were separated by two-dimensional difference gel electrophoresis (2D-DIGE) as described previously (De Haes et al., 2014). Samples were differentially labeled with either Cy3 or Cy5 fluorescent dyes (GE Healthcare). A possible dye bias was taken into account by integrating a dye swap into the experimental design. Two differentially labeled samples were pooled, an internal standard labeled with Cy2 was added, and the pooled samples were separated first based on pI and then based on MW. Between the two stages of separation, the proteins in the gel were reduced using dithiothreitol to break disulfide bonds and the formation of new disulfide bonds was blocked using iodoacetamide. Gels were scanned using an Ettan DIGE Imager (GE Healthcare), and DeCyder 7.0 (GE Healthcare) was used to detect significantly differential spots. To select the spots to excise, ANOVAs with false positive rate correction were used to determine differences between groups and potential interaction effects between bacterial strain and metformin.

Differential spots were excised from preparative gels (2 mg of protein from mixed samples per gel) using an automated spotpicker (GE Healthcare). excised gel plugs were washed with ultrapure water and subsequently dehydrated by treating them with an acetonitrile solution. These steps were repeated again, and the plugs were allowed to completely air dry, allowing them to efficiently take up the subsequently added trypsin digestion buffer (25 mM ammonium carbonate, 5% (vol/vol) acetonitrile, 100 ng of sequencing grade modified trypsin (Promega)). After incubation at 37°C overnight, the trypsinised peptides were collected, lyophilized and desalted. The resulting purified tryptic peptides were loaded on a Q-Exactive orbitrap (Thermo) and fragmented via high energy collision induced dissociation. To finally identify the proteins, the mass spectra from the orbitrap runs were analyzed using Peaks 7.5 (Bioinformatics Solutions Inc.). A parent mass error tolerance of 10 ppm was used and the fragment mass error tolerance was set at 0.02 Da. Additionally, one missed trypsin cleavage between peptides was tolerated and carbamidomethylation (C) and oxidation (M) were respectively selected as fixed and variable modifications. Using these settings, Peaks searched the curated Uniprot database for *E. coli* proteins.

Protein abundance estimates were *log*_*2*_ transformed and a linear model was fitted to the data to perform multiple univariate analysis. Significant differences in protein levels were determined using post hoc Tukey’s multiple comparison statistical test. Benjamini–Hochberg multiple comparison adjustment was applied with a FDR threshold of < 0.05. Transcription factor (TF) enrichment analysis was estimated using TF-gene association data from RegulonDB and by applying a hyper-geometric test. KEGG pathway and GO term enrichment was acquired using online DAVID enrichment analysis service. Enrichment was considered significant following Benjamini–Hochberg multiple comparison adjustment with a FDR threshold of < 0.05.

#### Bacterial Metabolomics

Bacterial cultures were prepared by using 500 μL of overnight bacterial culture to inoculate 50 mL of control liquid NGM and liquid NGM supplemented with metformin (50 mM) or IPTG (50, 100 μl). 3-4 independent biological replicates were prepared for each condition. Bacteria was grown at 25°C for 24 hours with constant shaking at 180 rpm. Cultures were then chilled on ice for 5 minutes before being centrifuged for 10 minutes at 6400 g, 4°C. Supernatant was removed except for 500 μL that was used to resuspend the bacterial pellet. Samples were then transferred to 5 mL tubes and were centrifuged as before. The supernatant was completely removed and tubes were flash frozen in liquid nitrogen. Samples were then stored at −80°C until metabolite extraction.

Metabolites were extracted by adding 1.6 mL of ice cold 100% HPLC grade methanol to each sample. Samples were kept on ice and were sonicated for 30 s at an amplitude of 5 microns. 1.1 mL of ice-cold internal standard solution (provided by HMT, diluted 2500-fold) was added to each sample and samples were vortexed for 30 s to mix thoroughly. 2 mL of extraction solution was transferred into 2ml tubes and centrifuged at 16,100 g for 20 minutes at 4°C. 1.6 mL of the resulting supernatant was divided into four filter units (provided by HMT and previously washed with double distilled water) and was spun down in a microcentrifuge at 9200 g until all sample had been filtered through (usually taking approximately 3 hours). All resulting filtrates from one sample were mixed and transferred into a new 2 mL tube. Samples were shipped to Human Metabolome Technologies (HMT), Inc. on dry ice for further processing and metabolomic analysis (Yamagata, Japan).

At HMT, samples were centrifuged and resuspended in 50 μL of ultrapure water immediately prior to measurement. Cationic metabolites were measured by CE- TOFMS in the positive ESI mode and anionic metabolites were measured by CE-QqQMS in the positive and negative ESI mode. Samples were diluted to improve the quality of analysis. Peaks detected in CE-TOFMS analysis were extracted using MasterHands ver.2.17.1.11 automatic integration software (Keio University) and those in CE-QqQMS analysis were extracted using MassHunter B.06.00 automatic integration software (Agilent Technologies) in order to obtain peak information including m/z, migration time (MT), and peak area. The peak area was then converted to relative peak area. The peaks were annotated based on the migration times in CE and m/z values determined by TOFMS. In addition, absolute quantification was performed for 116 metabolites including glycolytic and TCA cycle intermediates, amino acids, and nucleic acids. Metabolite concentrations were calculated by normalizing the peak area of each metabolite with respect to the area of the internal standard and by using standard curves, which were obtained by three-point calibrations. Metabolite concentrations for each sample were normalized by sample volume and OD_600_ of the original bacterial culture from which the sample was derived. Concentrations were *log*_*2*_ transformed and a linear model was fitted to the data for multiple univariate analysis. Significant differences in metabolite levels were estimated using post hoc Tukey’s multiple comparison statistical test. Benjamini-Hochberg multiple comparison adjustment was applied with an FDR threshold of < 0.05.

#### Bacterial Macromolecular Composition Analysis

Overnight bacterial cultures were centrifuged at 4,300 g for 15 minutes and cells were washed twice with PBS before being resuspended in a final volume of 1 mL PBS. OD_600_ measurements were taken for each sample. Samples were then sub-divided into 3 aliquots of equal volume (to be used for each type of macromolecular assay) and were centrifuged at 15,000 g for 5 minutes. The supernatant was removed and the bacterial pellet was flash frozen in liquid nitrogen and stored at −80°C until required.

To measure the total protein concentration, samples were kept on ice and pellets were resuspended in 150 μl B-PER reagent (Thermo Fisher Scientific). Samples were incubated at room temperature for 15 minutes with constant shaking at 700 rpm to lyse cells. Cellular debris was removed by centrifuging the resulting lysate at 15,000 g for 5 minutes at 4°C and transferring the supernatant to clean tubes. Protein concentrations were then determined via the Bradford assay. Total carbohydrate concentration was assayed by the anthrone method. Bacterial pellets were dissolved into anthrone solution in 70% H_2_SO_4_. Samples were heated for 20 minutes at 90°C. Once cooled, the lysate absorbance was measured at 620 nm, relative to a D-glucose standard curve. To measure total lipid concentration, lipids were extracted into organic solvent consisting of 7:11:0.1 chloroform:isopropanol:NP-40. Samples were homogenized in a bead beater (∼500 μm glass beads), and sonicated. Lysates were centrifuged at 16,000 x g, then the supernatants were dried by vacuum (Savant SpeedVac), and the final pellet was resuspended in 20 μL PBS, 0.05% Tween-20. Clarified samples were analyzed using Infinity Triglycerides Reagent (Thermo Scientific, #TR-22421) measuring the absorbance at 540 nm. The total concentration of each type of macromolecule was then normalized by the OD of the original sample. 6 independent biological replicates were included per condition. Statistical analysis was performed by two-way ANOVA using GraphPad Prism 6 software.

#### Nematode Metabolomics

Worms were grown on plates seeded with OP50 or test bacterial strains from the L1 stage at 20°C. Worms were transferred to control plates or plates supplemented with 50 mM metformin at the L4 stage using sterile PBS. On day 2 of adulthood, worms were collected and washed 3 times using sterile PBS in 2 mL microcentrifuge tubes. Supernatant was removed and the tube with the worm pellet was flash frozen in liquid nitrogen and stored at −80°C until metabolite extraction and analysis. Approximately 2000 worms were collected per sample for 3-4 independent biological replicates per condition.

Methods utilized here have been previously described and validated in *C. elegans* (Gao et al., 2017). To extract fatty acids (FA), samples were freeze-dried overnight and subsequently re-suspended in 500 μL of ice-cold 0.9% NaCl solution. A 5 mm steel bead was added to each tube and samples were lysed twice using a TissueLyser II (QIAGEN) (2.5 min, 30 beats/sec frequency). Samples were then tip sonicated twice (energy level: 40 Joule; output: 8 Watts) on ice. Protein quantification was performed via BCA assay. 150 μg worm protein lysate was transferred to a 4 mL FA-free glass vial, and 1 mL of freshly prepared 100% acetonitrile / 37% hydrochloric acid (4:1, v/v) was added to the lysate, together with deuterium-labeled internal standards (d5-C18:0 (5.04 nmol), d4-C24:0 (2.52 nmol), and d4-C26:0 (0.25 nmol)). Samples were vortexed for 5 s and hydrolysed by incubating at 90°C for 4 hours. After incubation, samples were cooled to room temperature, 2 mL hexane was added and samples were vortexed for 10 s. The upper layer was transferred to a FA-free glass vial and evaporated under a nitrogen stream at room temperature. 100 μL of hexane was added to each tube and samples were transferred to a Gilson Vial. ESI-MS analysis was carried out using an Acquity UPLC Binary Solvent manager (Waters, Milford MA) with an Acquity UPLC sample manager connected to a Quattro Premier XE mass spectrometer (Waters, Milford MA), run in the negative ESI mode. FA concentrations were calculated using a five-point calibration curve for C18:0, C24:0 and C26:0 (analytes). The calibration mixture contained different types of FA species (0, 25, 50, 100 and 200 μl) added to 50 μL of internal standard composed of d5-C18:0 (100.8 μM), d4-C24:0 (50.29 μM), and d4-C26:0 (5.06 μM). Standards were extracted and analyzed as described above. The input concentration for each FA was plotted against the ratio of the peak height of the analyte to the peak height of the corresponding internal standard. The resulting standard curve was used to calculate sample FA concentrations. The FA concentrations in each sample were then normalized by sample volume and protein concentration of worm lysate. Concentrations were *log*_*2*_ transformed and a linear model was fitted to the data. Significant changes in metabolite levels were estimated using multiple univariate analysis and post hoc Tukey’s multiple comparison statistical test. Benjamini-Hochberg multiple comparison adjustment was applied with an FDR threshold of < 0.05.

#### Nematode Lifespan Analysis

Experimental NGM plates were prepared by seeding with 100 μL of bacterial culture and leaving lawns to grow for 4 days at 20°C. To prevent progeny development, plates were supplemented with 5-fluoro-2′-deoxyuridine (FUdR, 30 μM) one day prior to use. To initiate the experiment (day 0), worms that had been age-synchronized via alkaline hypochlorite treatment were transferred to plates at the L4-stage, unless otherwise stated. For perhexiline treatment, worms were grown on plates supplemented with 2.5 mM perhexiline from the L1-stage and were additionally exposed to metformin from the L4-stage. For *acs-1* RNAi knockdown, worms were grown on RNAi bacteria from the L1-L4 stage and were then transferred onto OP50 for the remainder of the experiment. For *cco-1* RNAi knockdown, worms were grown on RNAi bacteria from egg-L4 stage and were then transferred onto OP50 for the remainder of the experiment. Worms were maintained at 20°C and were transferred to fresh plates every 4 days until day 12. Survival was monitored at regular time points and worms were scored as dead if they did not respond to touching with a platinum wire. Worms that exhibited severe vulva protrusion were censored. Statistical significance was estimated by the log rank test and Cox proportional hazards (CPH) analysis where appropriate using JMP 12 (SAS Institute). All lifespans represent pooled data from 2-3 independent experimental replicates (with the exception of Figures S2K, S2L, S3E, S4I, and S4J). Within each experimental replicate, 2-3 independent populations of approximately 30 worms (∼60-90 worms total) were included per condition. Associated statistics can be found in Table S1.

#### Nematode Fluorescence Microscopy

Experimental NGM plates were seeded with bacteria and supplemented with FUdR as for lifespan analysis and worms were transferred onto plates at the L4-stage. For experiments involving OXCT-1/C05C10.3 RNAi knockdown, worms were grown on RNAi bacteria from egg and were transferred onto OP50 at the L4 stage. For the quantification of transgenic reporter strain fluorescence, worms were maintained at 20°C and were imaged on day 2 of adulthood for all experiments with the exception of those involving the *Δcrp* pCrp bacterial strain where imaging took place on day 4 of adulthood. Worms were anesthetised with 1% levamisole on NGM plates and were imaged under a 63x objective using a Zeiss Axio Zoom V16 microscope system equipped with an AxioCam MRm camera operated by Zen 2 software (Zeiss). Either the GFP filterset (excitation: 450-490 nm; emission: 500-550 nm) or the RFP filterset (excitation: 559-585 nm; emission: 600-690 nm) was used depending on the strain being imaged. All images were exported in TIFF or CZI format and fluorescence levels were quantified using Volocity 5.2 software (PerkinElmer) run on a Surface tablet (Microsoft). The fluorescence intensity of individual worms was calculated as the pixel density of the entire cross-sectional area of the worm from which the pixel density of the background had been subtracted. 2 independent trials were carried out with a minimum of 15 worms imaged per condition per trial. If worms were imaged in groups (Figures S1C, S7K, and S7L) the fluorescence intensity was calculated automatically by setting a minimum threshold intensity that excluded the background. 3 independent trials were carried out with 1-2 groups of 8 worms imaged per condition per trial. Data was analyzed using R (R core team). Fluorescence intensity values were *log*_*2*_ transformed and multiple univariate analysis was performed using a linear model.

Confocal microscopy was carried out with the *Pdhs-3::dhs-3*::GFP and *Pvha-6*::mRFP-PTS1 strains in order to visualize lipid droplets and peroxisomal networks, respectively. L4-stage worms were transferred onto experimental plates and were maintained at 20°C until imaging on day 2 of adulthood. Worms were mounted onto 2% agarose pads and were anesthetised with 1% levamisole. Worms were imaged under a 63x oil-immersion objective using a Zeiss LSM 880 microscope system controlled by Zen Black software (Zeiss). Images were taken in the region of the anterior intestinal cells of the *Pdhs-3::dhs-3*::GFP reporter strain using the argon laser at 488 nm. Images were taken of the intestinal mid-section of the vha-6p::mRFP-PTS1 strain, in accordance with a previous study (Weir et al., 2017), using the DPSS 561-10 laser at 561 nm. 2-3 independent trials were carried out with a minimum of 11 worms imaged per condition.

#### Nematode RNA Sequencing

Experimental plates were seeded with 150 μL of bacterial culture and lawns were left to grow for 4 days at 20°C. Worms were age-synchronized by alkaline hypochlorite treatment and were transferred onto plates at the L4 stage. Approximately 1000 worms were transferred per condition for a total of 4 independent biological replicates. Worms were maintained at 20°C before collection on day 2 of adulthood. Worms were collected from plates in sterile nuclease free water and were transferred to 2 mL lysing matrix D tubes (MP Biomedicals) with lysing beads removed. Worms were then washed three times by letting the worms settle at the bottom of the tube, removing the supernatant and resuspending them in sterile nuclease free water. On the final wash, the supernatant was removed to leave worms in 100 μL of liquid. The lysing beads were added back to tubes with 400 μL of TRIzol (Zymo research) and after ten seconds of shaking, samples were flash frozen in liquid nitrogen and were then stored at −80°C until RNA extraction. To extract RNA, frozen samples were vortexed until thawed and then flash frozen in liquid nitrogen again. Samples were then vortexed for a total of 10 minutes with 30 s of chilling on ice every 2 minutes to prevent heating of samples. Samples were transferred to RNase free tubes and 1 volume of 100% ethanol was added. RNA was extracted using a Direct-zol RNA Miniprep kit (Zymo Research). The RNA concentration of each sample was measured using a NanoDrop 2000 spectrophotometer (Thermo Fisher Scientific) and a Qubit assay kit (Thermo Fisher Scientific). The RNA quality of each sample was measured using a 2100 BioAnalyzer (Aligent). All samples tested had RIN values of 9.8-10. Samples were shipped on dry ice to Genewiz, Inc (NJ, USA) for RNaseq.

RNA libraries were prepared using a NEBNext Ultra RNA library preparation kit with poly-A selection. Sequencing was performed on an Illumina HiSeq 2500 with approximately 16 million single-end 50bp reads generated per sample. Raw read quality was checked using FastQC 0.11.5 and contaminating sequence adapters were removed using Trimmomatic 0.36. Clean reads were aligned to the *C. elegans* reference genome WBcel235 using HISAT 2.0.5. Read counts were obtained at gene level using StringTie 1.3.3 with *C. elegans* WBcel235 Ensembl annotation v87. Transcription analysis was performed at the gene level using EdgeR. Only genes with counts per million (CPM) > 1 in all samples in at least one experimental group were considered for analysis. A generalized linear model was fitted to the data and differential gene expression was estimated. Batch adjustment was performed by incorporating an additional batch factor in the model. Differential gene expression was considered significant after Benjamini–Hochberg multiple comparison adjustment with a FDR threshold of < 0.05. Enrichment analysis was performed for GO terms and KEGG pathways, using the goana and kegga functions of the *limma* package with adjustment for gene length. Gene were mapped to annotations using their Entrez Gene IDs. Significance of KEGG pathway and GO term enrichment was determined following Benjamini–Hochberg multiple comparison adjustment with an FDR < 0.05. The “Longevity effect” could be more precisely stated as a “metformin induced and bacteria modulated lifespan extension effect” given that metformin supplementation only extends worm lifespan on OP50, but not on the metformin-resistant OP50-MR strain. Given these properties of the OP50-MR, we use it as a reference in identifying the transcriptional changes which can be associated both with the metformin induced nematode lifespan extension and its bacterial modulation by metformin sensitive OP50 strain. We accomplish this by subtracting the transcriptional changes observed in metformin treatment in worms incubated on OP50-MR from the corresponding changes observed in worms incubated in OP50. Therefore, the “longevity effect” is not a combination of metformin’s effects, but the difference of its effects between worms incubated on OP50 versus OP50-MR.

#### Four-way Host-Microbe-Drug-Nutrient Screen

Biolog phenotype microarray (PM) plates PM1 & PM2A containing carbon sources, PM3B containing nitrogen sources and PM4A containing sulfur and phosphorus sources were used in the screen. Caproic acid, Capric acid, 4-Hydroxy benzoic acid and 2-Hydroxy benzoic acid were excluded from analysis due to strong detrimental effects on bacterial growth. Liquid NGM was used as the based media instead of the Biolog IF-0a media provided by Biolog. Liquid NGM ± 50 mM metformin was supplemented with 1X tetrazolium dye and was inoculated with an overnight culture of OP50 at a final OD_600_ of 0.026. Plates were incubated for 24 hours at 37°C, 180 rpm and bacterial growth (via tetrazolium dye precipitation) was measured at 750 nm every 5 minutes using a Tecan Infinite M2000 microplate reader operated via Magellan V6.5 software (Tecan). 4 independent replicates were carried out per plate. Bacterial growth was estimated as the OD area under the curve (AUC) integral at 750 nm. AUC values were *log*_*2*_ transformed and were normalized by the bacterial growth values on NGM base media in the absence of nutrient sources. A linear model was then fitted to the data and multiple univariate analysis was performed. Significant effects of nutrient supplementation, metformin treatment and their interaction were estimated using post hoc Tukey’s multiple comparison statistical test. Benjamini–Hochberg multiple comparison adjustment was applied with an FDR threshold of < 0.05.

For measurement of *C. elegans Pacs-2*::GFP fluorescence, the nutrients in the wells of the Biolog plates were resuspended in 220 μL of molten NGM agar supplemented with 50 mM metformin and transferred to fresh 96-well plates. The agar was left to solidify for 30 minutes and plates were dried in a laminar flow hood for 20 minutes. 5 μL of overnight OP50 culture was added to each well and plates were again dried in a laminar flow hood for 2 hours. Plates were then incubated at 20°C for 4 days to allow bacterial lawns to grow. 5 worms were transferred to each well at the L4 stage of development. 6 μL of 2.5 mM FUdR was added to each well one day prior to transferring worms to prevent the development of progeny. Worms were maintained at 20°C for 30 hours. Worms were prepared for imaging by adding 10 μL of 1% levamisole to each well. Fluorescence microscopy was carried out under a 30x objective using a Leica M205FA microscope with a GFP filter set (excitation: 450-490 nm; emission: 500-550 nm) operated by LAS V4.0 software (Leica Microsystems). Images were taken of each well individually and files were exported in TIFF format. A total of 5 independent trials were carried out per plate. Fluorescence data was analyzed using Python *scikit-image* and *scikit-learn* libraries. TIFF images were converted from RGB to HSV color space for easy separation of color and brightness information. Individual worms in images were separated using adaptive thresholding, sobel filtering and watershed segmentation. Individual worm fluorescence intensity levels were then *log*_*2*_ transformed to allow relative comparisons to be made. Due to the segmented expression of the *Pacs-2*::GFP transgene throughout the worm, fluorescence intensity had a wide and non-normal distribution, with minimum and maximum values spanning the whole dynamic range captured in the image. In order to increase the analysis sensitivity to subtle changes in fluorescence intensity, the 90th quartile (Q90) of *log*_*2*_ transformed intensity distribution was used as a robust measure of maximum fluorescence intensity in individual worms. These estimates were normalized against the values obtained for worms maintained on negative control wells (NGM + 50 mM metformin with no nutritional supplement). No significant and measurable differences in *Pacs-2*::GFP levels by nutrient supplementation were observed in the absence of metformin treatment (data not shown). This enabled the relative increase or decrease in transgene expression levels caused by the presence of each nutritional supplement to be calculated. Multiple univariate analysis was performed with these values using a linear model and significance of fluorescence changes was determined after Benjamini–Hochberg multiple comparison adjustment with an FDR threshold of < 0.05.

Enrichment analysis of the data obtained from the four-way screen was performed in terms of KEGG pathways and metabolite classes for both bacteria and *C. elegans*. Metabolite class data was acquired from the EcoCyc database using *PathwayTools* and *pythoncyc* API with unique EcoCyc IDs of each supplement. KEGG pathway data was collected using the *E. coli* annotation database *org.EcK12.eg.db Bioconductor* package in R (R core team) and KEGG IDs provided by Biolog. Both metabolite class and KEGG pathway enrichment was estimated using a hypergeometric test. Term enrichments were considered significant following a Benjamini–Hochberg multiple comparison adjustment with an FDR threshold of < 0.05.

#### *Drosophila melanogaster* Lifespan Analysis

Flies were reared at standard larval density by transferring ∼18 μl of egg suspension into bottles. Eclosing adults were collected over a 12-h period, and allowed to mate for 48 h before sorting females at a density of 15 per vial, using 10 vials per condition.

For the agmatine lifespan experiments, SYA medium (5% sucrose, 10% yeast (MP Biomedicals), 1.5% agar, plus nipagin and propionic acid as anti-fungal agents; (Piper et al., 2014)) was supplemented with 0, 1, 2.5, 10 or 25 mM agmatine (from a 1 M stock in water, pH 5.5) while the food was ∼55°C. For the bacterial colonization lifespans, flies were made germ-free several generations prior to experiments by dechorionating eggs with 3%–5% bleach followed by washing in PBS. SYA food was used during development and mating, while lifespan assays were performed on chemically-defined or holidic medium (Piper et al., 2014) with minor modifications to obtain pH 6.5. For the modified chemically-defined medium, the pH of amino acid stocks was not adjusted, phosphate buffer at pH 6.5 was used (instead of acetate buffer), sucrose and propionic acid were omitted, and nipagin was used as sole anti-fungal agent. Modified holidic medium was supplemented with the appropriate concentration of metformin dissolved in water or IPTG at final concentration of 50 μM or equivalent volume of water as a control. All vials were spotted with 50 μl of overnight bacterial culture except those used for the germ-free group.

All flies were maintained at 25°C with 12 h light:12 h dark cycles and 60% humidity. Flies were transferred to fresh food and scored for survival every 2-3 days. Lifespan curves were plotted using JMP 12 (SAS Institute) and statistical significance was determined by the log rank test and by Cox Proportional Hazards analysis as appropriate. 1 trial was performed with a total of n∼150 flies per condition (10 independent vials containing 15 flies each).

##### *Drosophila melanogaster* Fecundity Assay

Flies treated with agmatine to day 7 were allowed to lay for 24 h. The total number of eggs laid was counted visually under a light microscope, then divided to give average fecundity per fly. Data are means of n = 10 vials with 15 females each per condition, with each vial representing an independent biological replicate. Statistical analysis was performed by one-way ANOVA using GraphPad Prism 6 software.

##### *Drosophila melanogaster* Body Weight Measurement

Flies treated with agmatine to day 14 were snap frozen in liquid nitrogen, and weighed in groups of 5 on a precision balance, then divided to give average body wet weight per fly. 8 independent biological replicates, each containing 5 flies. Statistical analysis was performed by one-way ANOVA using GraphPad Prism 6 software.

#### Refinement of Bacterial Metabolic Models

For microbial community modeling, we used a resource of 818 metabolic models of the human gut microbiota (AGORA resource, version 1.02, (Magnúsdóttir et al., 2017)). We extensively manually curated models from the AGORA resource to remove inconsistencies introduced in the draft reconstruction process that led to wrong annotations of reaction reversibility and energy generation through thermodynamically infeasible futile cycles in community modeling based on the originally published models (Graspeuntner et al., 2019). Since AGORA models did not contain transporters for agmatine, we queried TransportDB (http://www.membranetransport.org/transportDB2/index.html) for known agmatine transporters and included them based on the detection of homologs to reported sequences in Transport DB in the corresponding bacterial models. More precisely, a BLAST search of known agmatine transporter proteins against the genome sequences of the bacterial species within the AGORA collection was performed (i.e., tblastn). BLAST hits with a bit-score above 50 (empiric cut-off for transporter genes) and a query coverage equal or above 75% were considered as evidence for the existence of the corresponding agmatine transporter. In total, we identified at least one agmatine transporter in 257 species. Since SpeA, one of the two agmatine-producing enzymes in *E. coli*, is reported to be located in the cell wall (*cf.* https://ecocyc.org/gene?orgid=ECOLI&id=EG10959), we additionally searched for homologs of this gene in genomes of species of the AGORA resource using a bitscore cut-off of 200 (empiric cut-off for non-transporter genes). We identified homologs of *speA* in 124 species for which we added an extracellular agmatine-producing reaction from arginine based on the cytosolic “ARGDC” reaction contained in the corresponding models (with the ID ARGDC_EXT). Additionally, an agmatine outflow reaction was added for each species in which either an agmatine transporter or an agmatine biosynthetic gene was detected. Subsequently, we tested production of agmatine across all bacterial models using flux balance analysis by maximizing agmatine production assuming the Kiel Diet (see below). We found that 182 out of 812 models were able to produce agmatine. We tested the ability of the models of the AGORA resource to grow on the Kiel diet. To this end, we constrained each model with this diet and maximized bacterial biomass production. We assumed a cut-off of a minimal biomass production of 0.01 mmol/gM/d for growth (see the section “In silico Prediction of Microbial Agmatine Production” for details on the unit of measurement). Based on this cut-off, we identified 58 out of 818 AGORA models that were not able to grow and thus excluded from further analysis.

#### Derivation of the Kiel Cohort Diet

For microbial community modeling, detailed information on the nutritional compounds available to the microbial community is required. Here we took advantage of the availability of detailed dietary information of the individual participants from the Kiel cohort from the EPIC food frequency questionnaire (Brandstetter et al., 1999) that allows to derive detailed information on the molecular composition of the diet of each participant that can be used as input for community modeling. In a first step, we mapped dietary compounds present in the EPIC data to the corresponding exchange compound present in the AGORA models (see Table S5F). Subsequently, the weight of the corresponding compounds was converted to millimoles using information on the molecular weight of each compound. Subsequently, molecular concentrations were divided by 200 to obtain nutrients available per gram of bacterial biomass in the gut microbiome. We noted that the EPIC data contained only limited information on nitrogen-rich compounds such as nucleotides and polyamines. Thus, we expanded the molecular composition of the diets of each participant through information on the composition of these compounds. To this end, we made use of information on the consumption of different types of food items from the EPIC data (measured in gram across a list of 140 hierarchically ordered food items).

For nucleotides (purines and pyrimidines), we used various literature sources as reference (Table S5F). We matched food items from the EPIC data to the foods in the database and calculated the average for each group/subgroup (in the EPIC data). In case of missing information for a food item (e.g., beer), we used alternative sources or available information from websites. If we were not able to find alternative information, we used average values from the corresponding food group within the EPIC data as source. Since measurements only indicated total amounts of each type of nucleotide (e.g., adenosine), we splitted the corresponding molecular concentration into the different corresponding molecular compounds (e.g., adenosine and desoxyadenosine). Since no information on pyrimidine content of food items was available we extrapolated values from purines to pyrimidines. Thus, we assumed that the molecular concentration of the purine adenosine equates the molecular concentration of thymidine and uracil combined and that the molecular concentration of guanosine equals that of cytosine. For more details on the assumptions used for deriving molecular quantities of individual compounds, please see Table S5F. For polyamines, we used measured quantities of cadaverine, putrescine, spermidine and spermine of typical food items from the literature (Table S5F). The quantity of ornithine in food items has been only poorly reported so far. Thus, we used information on reported ornithine content for food items for which we also had data on other polyamines available to derive a median ornithine to polyamine ratio. We found that the median ornithine to polyamine ratio for all foods with complete data available was 3.28. Thus, for all food items without data, we extrapolated ornithine content from the content of cadaverine, putrescine, spermidine and spermine through multiplication of total molecular concentrations of these compounds with the factor 3.28.

To derive participant-specific diets, we determined the molecular composition of their dietary uptake based on details provided by the food frequency questionnaire with the additions reported above. To make diets comparable across participants, we normalized them according to the reported caloric value of each diet to the median caloric value across all participants (8799 kJ). Moreover, the bacterial metabolic models from the AGORA resource were originally reconstructed and tested on a predefined “Western diet” or similarly defined diets (Magnúsdóttir et al., 2017). To avoid problems due to individual compounds not covered by our derived diet on the metabolic capacity of individual bacterial models, we combined our derived diet with the originally defined Western diet. To this end, we derived participant-specific diets by reducing the influx of compounds provided by the original Western diet to 10% of their original value and supplementing 90% of the recorded values of dietary intake of each compound for each participant from the Kiel cohort. Additionally, we retained the original constraints on the inflow of phosphate, copper, manganese, zinc, iron(3+) and chloride as we found these compounds to strongly limit growth for individual bacterial models. Predicted agmatine production in metformin users also remained significantly increased compared to healthy obese controls, when using 1% of the Western diet (99% diet from the participants) or 100% of the Western diet (0% recorded diet from the participants) on the Kiel cohort (Table S5M). Likewise, for the other cohorts for which we did not have specific dietary data available, we used the average uptake across all participants from the Kiel cohort (see Table S5F for the molecular composition) combined with 10% Western diet.

The derived dietary input for each participant has been deposited in the PopGen Biobank (Schleswig-Holstein, Germany) and is available via a structured application procedure (https://www.uksh.de/p2n/Information+for+Researchers.html).

#### Derivation of Community Composition from Metagenomics Data

Previously published metagenomic sequences, obtained from feces of type 2 diabetic patients distinct for metformin treatment and healthy controls, were obtained from the European Nucleotide database and the Sequence Read Archive (see section Cohort Information for Accession Numbers and Metadata). The raw read data was extracted, merged sample wise and quality controlled for adaptor contamination and base call qualities. Adaptor sequences with an overlap of ≥ 3 bp as well as base calls with a Phred+33 quality score of < 30 were trimmed from the 3′ ends of reads using cutadapt (version 1.12). As adaptor sequences, we employed Illumina’s Nextera transposon sequence (CTGTCTCTTATACACATCT) together with the reverse complements of the TruSeq forward (CCGAGCCCACGAGACNNNNNNNNATCTCGTATGCCGTCTTCTGCTTG) and reverse primers (GACGCTGCCGACGANNNNNNNNGTGTAGATCTCGGTGGTCGCCGTATCATT).

Subsequently, reads were quality controlled via prinseq lite (version 0.20.4) by a sliding window approach with step size 5 bp, window size 10 bp, mean base quality < 30 and a minimum length filter that discarded any reads shorter than 50 bp (Spanish cohort) or 35 bp (Danish and Swedish cohorts) after all other quality controlling steps. In order to filter out host sequences, remaining sequences were mapped to the human reference genome (hg38), as released in December 2013 in the version of the University of California Santa Cruz, including unplaced and unlocalized parts. The unmapped remainder of reads were than mapped against the reconstructed bacterial genomes published in the AGORA set (version 1.02, obtained from https://webdav-r3lab.uni.lu/public/msp/AGORA/genomes/ in March 2018). The number of reads mapping to each AGORA species can be found in the Mendeley data archive (https://data.mendeley.com/datasets/crmtpmd622/draft?a=ef347ccd-7532-44b0-8925-d2c04a71b419). Differential abundance of bacterial species between metformin treated versus untreated was tested via generalized linear models as implemented in the R-package “DESeq2.” Statistical evaluation was performed via the two-sided Wilcoxon rank-sum test without continuity correction and via the R-package “DESeq2.”

#### *In silico* Prediction of Microbial Agmatine Production

Please note that while in constraint-based metabolic models the usual unit of measurement is mmol per gram dry-weight per hour we here use mmol per gram microbiome (i.e., full weight of the bacteria in the gut) by normalizing dietary uptake to the amount of compounds available per gram of microbiota in the colon (estimated total weight of 200 g) per day. Thus, the unit of measurement of metabolic fluxes for the community simulations is mmol per gram microbiota per day (mmol/gM/d). In order to predict microbial production of agmatine, we derived microbial community models for each patient in the individual cohorts (Graspeuntner et al., 2019). To this end, we initially determined the microbial species present in each participant’s fecal microbiome. For 16 s rRNA profiling data, 16S rRNA gene reads for each participant were mapped against the 16S rRNA genes of species from the AGORA resource and the closest matching bacterial species above a similarity threshold of 97% was used (*cf.* (Graspeuntner et al., 2019)). For metagenomics data, metagenomic reads were mapped against the genomes of species from the AGORA resource as described above. After mapping reads to species, abundances were normalized to relative abundances for each participant to a sum of 1 across all mapped species. For 16S rRNA profiling data we assumed presence of a species for a relative abundance cut-off of ≥ 0.1% of total reads and an abundance cut-off of ≥ 0.01% for metagenomic data due to the lower read depth of 16S rRNA profiling data compared to metagenomics data. Subsequently, the models representing the species contained in each participant’s microbiome were joined together in a common compartment. In order to enforce the species composition detected in each participant, biomass outflow reactions of the individual bacterial models were blocked and an additional biomass outflow reaction was introduced that consumed the biomass of all present bacteria in the relative proportion in which each bacterium was detected in the microbiota. Additionally, we used coupling constraints to connect flux through each reaction in a species to a minimal biomass production for that species (Graspeuntner et al., 2019). Models were constrained with either the Kiel diet as reported above (for testing agmatine production in the Spanish, Swedish and Danish cohort) or the respective diet of each individual participant (for testing the influence of individual diets on agmatine production in the Kiel cohort). For the Kiel cohort we moreover tested the impact of using different relative compositions of the original Western diet versus the patient-specific diet (Table S5L).

To predict agmatine production capacity for the thus reconstructed microbial community of each participant, we used flux balance analysis with concomitant maximization of agmatine outflow and minimization of total flux as objective function using the R-package sybil (Gelius-Dietrich et al., 2013). The objective function coefficient for agmatine production was one and −10^−6^ multiplied with the absolute sum of flux for flux minimization (Graspeuntner et al., 2019). Predicted agmatine production capacity for each participant can be found in Supplementary Tables S5G-J. Species-specific agmatine production was determined by extracting the agmatine production of individual bacterial strains from metformin-treated patients from each cohort from the optimization results and summing across all bacterial models belonging to the same species. Likewise, side-products of agmatine production were obtained from metformin-treated patients from the Kiel cohort. In order to exclude that increased agmatine production in metformin-treated patients was due to differences in dietary habits between both groups, we repeated community simulations for the dietary input of each patient for the individual microbial composition of all diabetic patients. We found that for each individual patient’s diet, median predicted agmatine production capacity by microbiota of metformin-treated patients was always higher than that of non-metformin-treated patients. To investigate the influence of individual dietary compounds on agmatine production capacity, we repeated optimizations for metformin-treated type 2 diabetic patients from the Kiel cohort assuming the Kiel diet as base diet. For each participant and each dietary compound reported in the EPIC data, we tested the impact of supplying 1 mmol of the corresponding compound on maximal agmatine production (Table S5B).

It has to be noted that in our modeling approach we used the diet of each participant as input, while we used fecal microbiota as representative microbial community composition. While dietary absorption takes place mostly in the small intestine with a smaller contribution by the larger intestine (Kiela and Ghishan, 2016), the fecal microbiota mostly reflects the microbial composition of the large intestine. While peak concentrations of metformin have been observed in the jejunum (middle part of the small intestine), also around 30% remain unabsorbed and enter the large intestine (Kiela and Ghishan, 2016) affecting the microbial community there. Studies specifically investigating the microbiota of the small intestine report the presence of *Escherichia* and *Citrobacter* species (Sundin et al., 2017) which our modeling approach predicts to be potent producers of agmatine (Figure 5C). Thus, while our modeling approach most specifically reflects the diet available to the microbial community in the small intestine, for which no patient-specific data on microbiota composition is available, the most important producers of agmatine which we observe in the microbial communities of the large intestine are nevertheless present in the small intestine. Moreover, we have repeated community optimization for the Kiel cohort while removing compounds according to their absorption in the small intestine by subtracting from the diet of each participant for each compound the fraction of that compound being absorbed in the small intestine (Cohn et al., 2010, Elmadfa and Leitzmann, 2015). We find that all reported significant differences in the individual subgroups of the Kiel cohort remain significant when accounting for absorption (Table S5M).

#### Metabolic Modeling of *Escherichia coli* OP50

For metabolic modeling of *Escherichia coli* OP50 we derived the molecular composition of Nematode Growth Medium (NGM) according to the respective Cold Spring Harbor Protocol (http://cshprotocols.cshlp.org/content/2008/10/pdb.rec11474.full). The composition of peptone was based on BD Bacto Peptone (BD Bionutrients Technical Manual BD Biosciences – Advanced Bioprocessing, 4th edition https://www.bd.com/documents/guides/user-guides/DS_CM_Bionutrients-technical-manual_UG_EN.pdf) and the composition of BD Bacto Agar (Difco & BBL Manual, 2nd Edition https://www.bd.com/europe/regulatory/Assets/IFU/Difco_BBL/281230.pdf). We assumed unlimited oxygen supply. The pH value was obtained from the media published on http://protocols.mmml.nl/index.php/protocols2/c-elegans/elegans-media. The quantity of iron was evenly split between Fe^2+^ and Fe^3+^, while the quantity of glutamine was inferred from the quantity of glutamate. The full composition of the NGM medium is provided in Table S5A. To test which compounds could theoretically increase the production of agmatine in *E. coli* OP50 when supplemented to the growth medium we used flux balance analysis on a genome scale model of this strain (Zimmermann et al., 2019) to predict the maximum agmatine production capacity on NGM medium supplemented with different compounds. To this end, the derived NGM diet (see above) was extended by an additional inflow (+ 5 mmol/L) of each compound of interest. The increased agmatine production capacity was calculated as the predicted agmatine yield with compound supplementation divided by the predicted yield under NGM medium alone (Table S5B).

### Quantification and Statistical Analysis

#### General

Data was considered statistically significant when p < 0.05, one-way ANOVA, two-way ANOVA or Benjamini-Hochberg FDR < 0.05 as indicated in the figure, figure legend or experimental methods. Asterisks denote corresponding statistical significance ^∗^p < 0.05; ^∗∗^p < 0.01; ^∗∗∗^p < 0.001. Data is presented as the mean ± SD or mean ± SE where appropriate from at least 3 independent biological replicates, unless stated otherwise in figures, figure labels or experimental methods. Statistical analysis was performed using GraphPad Prism 6 software, log rank test in JMP 12 software (SAS Institute), linear modeling and ANOVA in R, as indicated.

#### High-Throughput Screens

Data analysis was performed using the R statistical analysis software package v3.5.0 (https://www.r-project.org) or Python v2.7.15 (https://www.python.org) unless stated otherwise. Linear modeling/regression was accomplished using R base function “lm,” and function “glht” from “multcomp” package. Data handling and plotting was performed using R “tidyverse” packages (https://www.tidyverse.org).

To test whether an increase in agmatine production capacity due to the specific nutrient supplementation was associated with increased growth of *E. coli* OP50 in Metformin-containing medium (Table S5D), we compared the predicted agmatine production capacity with the growth phenotypes as measured with BIOLOG C-source plates (see section “Four-way Host-Microbe-Drug-Nutrient Screen”). The association between predictions and measured growth (quantified as area under curve) was analyzed using linear mixed effect models within R and the package ‘lme4’ (version 1.1-19: The predicted agmatine production capacity was considered as fixed factor that influences growth. Intercepts for each replicate were defined as random effect to account for potential batch-effects between BIOLOG plates. To obtain p values, likelihood ratio tests of the full model against the control model without time as fixed effect of interest were performed. Visual analyses of residual plots revealed no obvious deviation of homoscedasticity or normality.

Similar to the effect on bacterial growth, we further investigated the association of *Pacs-2*::GFP fluorescence in *C. elegans* in different supplemented nutrient growth environments with the predicted agmatine production capacity of the associated *E. coli* OP50 population (Table S5C). A mixed ANOVA was used to analyze the association of agmatine production capacity with *Pacs-2*::GFP fluorescence while considering the replicate identity as repeated-measures.

#### Statistical Testing of Metformin-associated Differences in Agmatine Production from Human Cohorts

Differences in agmatine production between cohorts were tested using the Wilcoxon rank-sum test. Using phenotypic data available for the Kiel cohort, we tested the influence of 30 phenotypic parameters including body measures, blood chemistry, disease status and medication on predicted agmatine production. Since the groups within this cohort significantly differed in body mass index (BMI), we used partial spearman correlation by correlating predicted agmatine production of each participant against the individual phenotypic parameters while controlling for BMI. After correcting for multiple testing using false discovery rate control, we found that metformin treatment (FDR, p = 2.4x10^−4^), type 2 diabetic status (FDR, p = 1.3x10^−3^), age (FDR, p = 1.3x10^−2^), anti-hypertensive medication (FDR, p = 1.2x10^−2^), gender (FDR, p = 2.7x10^−2^) and coronary heart disease status (FDR, p = 3.7x10^−2^) was significantly associated with agmatine production. Also controlling for age, only metformin treatment (FDR, p = 4.2x10^−4^) and type 2 diabetic status (FDR, p = 2.8x10^−3^) remained significant. After removing metformin-treated patients from the cohort, type 2 diabetic status was not significantly associated with agmatine production anymore, thus excluding a primary influence of type 2 diabetic status on agmatine production alone. All interactions for the individual tests are provided in Table S5H.

For the longitudinal Spanish cohort, linear mixed effect (LME) models were used to investigate temporal changes in the microbiome’s capacity to produce agmatine during metformin treatment and placebo administration, respectively. Time (months of treatment) was considered as the fixed factor of interest. The initial levels of the agmatine production capacity (intercepts) for each patient was defined as random effect. LME-models were fitted using the R-package “lme4” (version 1.1-19). To obtain p values, likelihood ratio tests of the full model against the control model without time as fixed effect of interest were performed. Visually analyses of residual plots revealed no obvious deviation of homoscedasticity or normality.

### Data and Code Availability

*C. elegans* RNA sequencing data is available at ArrayExpress: E-MTAB-7272. Additional data associated with this paper has been deposited at Mendeley Data at http://data.mendeley.com/login?redirectPath=/datasets/crmtpmd622/draft?a=ef347ccd-7532-44b0-8925-d2c04a71b419. Computer code used in this study is available from GitHub: https://github.com/CabreiroLab/4-way_paper.

## Supplemental Information

Table S1. *C. elegans* and *D. melanogaster* Lifespan Data, Related to Figures 1–4, 7, S2–S5, and S7

Table S2. Four-Way Host-Microbe-Drug-Nutrient Screen Data, Related to Figures 1 and S1

Table S3. *E. coli* Proteomics Data, Related to Figure 2

Table S4. *E. coli* Metabolomics Data, Related to Figures 4, S4, and S5

Table S5. *E. coli* and Human Microbiota Metabolic Modeling of Agmatine Production Data, Related to Figures 5 and S5

Table S6. *C. elegans* RNA-Seq Data, Related to Figures 6 and S6

Table S7. *C. elegans* Fatty Acid Metabolomics Data, Related to Figures 6, 7, and S7

Table S8. List of Primers Used in This Study, Related to STAR Methods

Video Abstract

## References

[bib1] Ackerman D., Gems D. (2012). Insulin/IGF-1 and hypoxia signaling act in concert to regulate iron homeostasis in Caenorhabditis elegans. PLoS Genet..

[bib2] Barzilai N., Crandall J.P., Kritchevsky S.B., Espeland M.A. (2016). Metformin as a Tool to Target Aging. Cell Metab..

[bib3] Bauer P.V., Duca F.A., Waise T.M.Z., Rasmussen B.A., Abraham M.A., Dranse H.J., Puri A., O’Brien C.A., Lam T.K.T. (2018). Metformin Alters Upper Small Intestinal Microbiota that Impact a Glucose-SGLT1-Sensing Glucoregulatory Pathway. Cell Metab..

[bib4] Brandstetter B.R., Korfmann A., Kroke A., Becker N., Schulze M.B., Boeing H. (1999). Dietary habits in the German EPIC cohorts: food group intake estimated with the food frequency questionnaire. European Investigation into Cancer and Nutrition. Ann. Nutr. Metab..

[bib5] Burkewitz K., Morantte I., Weir H.J.M., Yeo R., Zhang Y., Huynh F.K., Ilkayeva O.R., Hirschey M.D., Grant A.R., Mair W.B. (2015). Neuronal CRTC-1 governs systemic mitochondrial metabolism and lifespan via a catecholamine signal. Cell.

[bib6] Cabreiro F., Au C., Leung K.Y., Vergara-Irigaray N., Cochemé H.M., Noori T., Weinkove D., Schuster E., Greene N.D., Gems D. (2013). Metformin retards aging in C. elegans by altering microbial folate and methionine metabolism. Cell.

[bib7] Chubukov V., Gerosa L., Kochanowski K., Sauer U. (2014). Coordination of microbial metabolism. Nat. Rev. Microbiol..

[bib8] Cohn J.S., Kamili A., Wat E., Chung R.W., Tandy S. (2010). Dietary phospholipids and intestinal cholesterol absorption. Nutrients.

[bib9] Datsenko K.A., Wanner B.L. (2000). One-step inactivation of chromosomal genes in Escherichia coli K-12 using PCR products. Proc. Natl. Acad. Sci. USA.

[bib10] David L.A., Maurice C.F., Carmody R.N., Gootenberg D.B., Button J.E., Wolfe B.E., Ling A.V., Devlin A.S., Varma Y., Fischbach M.A. (2014). Diet rapidly and reproducibly alters the human gut microbiome. Nature.

[bib11] DeCorby A., Gásková D., Sayles L.C., Lemire B.D. (2007). Expression of Ndi1p, an alternative NADH:ubiquinone oxidoreductase, increases mitochondrial membrane potential in a C. elegans model of mitochondrial disease. Biochim. Biophys. Acta.

[bib56] De Haes W., Frooninckx L., VanAssche R., Smolders A., Depuydt G., Billen J., Braeckman B.P., Schoofs L., Temmerman L. (2014). Metformin promotes lifespan through mitohormesis via the peroxiredoxin PRDX-2. Proc Natl Acad Sci U S A.

[bib12] Elmadfa I., Leitzmann C. (2015). Ernährung des Menschen.

[bib13] Fischbach M.A. (2018). Microbiome: Focus on Causation and Mechanism. Cell.

[bib14] Forslund K., Hildebrand F., Nielsen T., Falony G., Le Chatelier E., Sunagawa S., Prifti E., Vieira-Silva S., Gudmundsdottir V., Pedersen H.K., MetaHIT consortium (2015). Disentangling type 2 diabetes and metformin treatment signatures in the human gut microbiota. Nature.

[bib15] Gao A.W., Chatzispyrou I.A., Kamble R., Liu Y.J., Herzog K., Smith R.L., van Lenthe H., Vervaart M.A.T., van Cruchten A., Luyf A.C. (2017). A sensitive mass spectrometry platform identifies metabolic changes of life history traits in C. elegans. Sci. Rep..

[bib16] Garcia-Gonzalez A.P., Ritter A.D., Shrestha S., Andersen E.C., Yilmaz L.S., Walhout A.J.M. (2017). Bacterial Metabolism Affects the C. elegans Response to Cancer Chemotherapeutics. Cell.

[bib17] Gelius-Dietrich G., Desouki A.A., Fritzemeier C.J., Lercher M.J. (2013). Sybil--efficient constraint-based modelling in R. BMC Syst. Biol..

[bib18] Gonzalez P.S., O’Prey J., Cardaci S., Barthet V.J.A., Sakamaki J.I., Beaumatin F., Roseweir A., Gay D.M., Mackay G., Malviya G. (2018). Mannose impairs tumour growth and enhances chemotherapy. Nature.

[bib19] Graspeuntner S., Waschina S., Kunzel S., Twisselmann N., Rausch T.K., Cloppenborg-Schmidt K., Zimmermann J., Viemann D., Herting E., Gopel W. (2019). Gut dysbiosis with Bacilli dominance and accumulation of fermentation products precedes late-onset sepsis in preterm infants. Clin. Infect. Dis..

[bib20] Hilbert Z.A., Kim D.H. (2018). PDF-1 neuropeptide signaling regulates sexually dimorphic gene expression in shared sensory neurons of *C. elegans*. eLife.

[bib21] Ho C.L., Tan H.Q., Chua K.J., Kang A., Lim K.H., Ling K.L., Yew W.S., Lee Y.S., Thiery J.P., Chang M.W. (2018). Engineered commensal microbes for diet-mediated colorectal-cancer chemoprevention. Nat. Biomed. Eng..

[bib22] Hussey R., Stieglitz J., Mesgarzadeh J., Locke T.T., Zhang Y.K., Schroeder F.C., Srinivasan S. (2017). Pheromone-sensing neurons regulate peripheral lipid metabolism in Caenorhabditis elegans. PLoS Genet..

[bib23] Karlsson F.H., Tremaroli V., Nookaew I., Bergström G., Behre C.J., Fagerberg B., Nielsen J., Bäckhed F. (2013). Gut metagenome in European women with normal, impaired and diabetic glucose control. Nature.

[bib24] Kiela P.R., Ghishan F.K. (2016). Physiology of Intestinal Absorption and Secretion. Best Pract. Res. Clin. Gastroenterol..

[bib57] Kim D., Langmead B., Salzberg S.L. (2015). HISAT: a fast spliced aligner with low memory requirements. Nat Methods.

[bib25] Kim H.E., Grant A.R., Simic M.S., Kohnz R.A., Nomura D.K., Durieux J., Riera C.E., Sanchez M., Kapernick E., Wolff S. (2016). Lipid Biosynthesis Coordinates a Mitochondrial-to-Cytosolic Stress Response. Cell.

[bib26] Krawczak M., Nikolaus S., von Eberstein H., Croucher P.J., El Mokhtari N.E., Schreiber S. (2006). PopGen: population-based recruitment of patients and controls for the analysis of complex genotype-phenotype relationships. Community Genet..

[bib27] Kundu P., Blacher E., Elinav E., Pettersson S. (2017). Our Gut Microbiome: The Evolving Inner Self. Cell.

[bib28] Le Chatelier E., Nielsen T., Qin J., Prifti E., Hildebrand F., Falony G., Almeida M., Arumugam M., Batto J.M., Kennedy S., MetaHIT consortium (2013). Richness of human gut microbiome correlates with metabolic markers. Nature.

[bib29] Lee S.J., Murphy C.T., Kenyon C. (2009). Glucose shortens the life span of C. elegans by downregulating DAF-16/FOXO activity and aquaporin gene expression. Cell Metab..

[bib30] Li H., Handsaker B., Wysoker A., Fennell T., Ruan J., Homer N., Marth G., Abecasis G., Durbin R., 1000 Genome Project Data Processing Subgroup (2009). The Sequence Alignment/Map format and SAMtools. Bioinformatics.

[bib31] Lloyd-Price J., Mahurkar A., Rahnavard G., Crabtree J., Orvis J., Hall A.B., Brady A., Creasy H.H., McCracken C., Giglio M.G. (2017). Strains, functions and dynamics in the expanded Human Microbiome Project. Nature.

[bib32] Love M.I., Huber W., Anders S. (2014). Moderated estimation of fold change and dispersion for RNA-seq data with DESeq2. Genome Biol..

[bib33] Ma D.K., Vozdek R., Bhatla N., Horvitz H.R. (2012). CYSL-1 interacts with the O2-sensing hydroxylase EGL-9 to promote H2S-modulated hypoxia-induced behavioral plasticity in C. elegans. Neuron.

[bib34] Magnúsdóttir S., Heinken A., Kutt L., Ravcheev D.A., Bauer E., Noronha A., Greenhalgh K., Jäger C., Baginska J., Wilmes P. (2017). Generation of genome-scale metabolic reconstructions for 773 members of the human gut microbiota. Nat. Biotechnol..

[bib35] Maier L., Pruteanu M., Kuhn M., Zeller G., Telzerow A., Anderson E.E., Brochado A.R., Fernandez K.C., Dose H., Mori H. (2018). Extensive impact of non-antibiotic drugs on human gut bacteria. Nature.

[bib36] Müller N., Schulte D.M., Türk K., Freitag-Wolf S., Hampe J., Zeuner R., Schröder J.O., Gouni-Berthold I., Berthold H.K., Krone W. (2015). IL-6 blockade by monoclonal antibodies inhibits apolipoprotein (a) expression and lipoprotein (a) synthesis in humans. J. Lipid Res..

[bib37] Onken B., Driscoll M. (2010). Metformin induces a dietary restriction-like state and the oxidative stress response to extend C. elegans Healthspan via AMPK, LKB1, and SKN-1. PLoS ONE.

[bib38] Piper M.D., Blanc E., Leitão-Gonçalves R., Yang M., He X., Linford N.J., Hoddinott M.P., Hopfen C., Soultoukis G.A., Niemeyer C. (2014). A holidic medium for Drosophila melanogaster. Nat. Methods.

[bib39] Pryor R., Cabreiro F. (2015). Repurposing metformin: an old drug with new tricks in its binding pockets. Biochem. J..

[bib40] Puchalska P., Crawford P.A. (2017). Multi-dimensional Roles of Ketone Bodies in Fuel Metabolism, Signaling, and Therapeutics. Cell Metab..

[bib41] Qi B., Han M. (2018). Microbial Siderophore Enterobactin Promotes Mitochondrial Iron Uptake and Development of the Host via Interaction with ATP Synthase. Cell.

[bib58] Robinson M.D., McCarthy D.J., Smyth G.K. (2010). edgeR: a Bioconductor package for differential expression analysis of digital gene expression data. Bioninformatics.

[bib42] Rothschild D., Weissbrod O., Barkan E., Kurilshikov A., Korem T., Zeevi D., Costea P.I., Godneva A., Kalka I.N., Bar N. (2018). Environment dominates over host genetics in shaping human gut microbiota. Nature.

[bib43] Satishchandran C., Boyle S.M. (1986). Purification and properties of agmatine ureohydrolyase, a putrescine biosynthetic enzyme in Escherichia coli. J. Bacteriol..

[bib44] Schmidt T.S.B., Raes J., Bork P. (2018). The Human Gut Microbiome: From Association to Modulation. Cell.

[bib45] Schmieder R., Edwards R. (2011). Quality control and preprocessing of metagenomic datasets. Bioinformatics.

[bib46] Scott T.A., Quintaneiro L.M., Norvaisas P., Lui P.P., Wilson M.P., Leung K.Y., Herrera-Dominguez L., Sudiwala S., Pessia A., Clayton P.T. (2017). Host-Microbe Co-metabolism Dictates Cancer Drug Efficacy in C. elegans. Cell.

[bib47] Shin N.R., Lee J.C., Lee H.Y., Kim M.S., Whon T.W., Lee M.S., Bae J.W. (2014). An increase in the Akkermansia spp. population induced by metformin treatment improves glucose homeostasis in diet-induced obese mice. Gut.

[bib48] Slack C., Foley A., Partridge L. (2012). Activation of AMPK by the putative dietary restriction mimetic metformin is insufficient to extend lifespan in Drosophila. PLoS ONE.

[bib49] Sundin O.H., Mendoza-Ladd A., Zeng M., Diaz-Arévalo D., Morales E., Fagan B.M., Ordoñez J., Velez P., Antony N., McCallum R.W. (2017). The human jejunum has an endogenous microbiota that differs from those in the oral cavity and colon. BMC Microbiol..

[bib50] Weir H.J., Yao P., Huynh F.K., Escoubas C.C., Goncalves R.L., Burkewitz K., Laboy R., Hirschey M.D., Mair W.B. (2017). Dietary Restriction and AMPK Increase Lifespan via Mitochondrial Network and Peroxisome Remodeling. Cell Metab..

[bib51] Wu L., Zhou B., Oshiro-Rapley N., Li M., Paulo J.A., Webster C.M., Mou F., Kacergis M.C., Talkowski M.E., Carr C.E. (2016). An Ancient, Unified Mechanism for Metformin Growth Inhibition in C. elegans and Cancer. Cell.

[bib52] Wu H., Esteve E., Tremaroli V., Khan M.T., Caesar R., Mannerås-Holm L., Ståhlman M., Olsson L.M., Serino M., Planas-Fèlix M. (2017). Metformin alters the gut microbiome of individuals with treatment-naive type 2 diabetes, contributing to the therapeutic effects of the drug. Nat. Med..

[bib53] You C., Okano H., Hui S., Zhang Z., Kim M., Gunderson C.W., Wang Y.P., Lenz P., Yan D., Hwa T. (2013). Coordination of bacterial proteome with metabolism by cyclic AMP signalling. Nature.

[bib54] Zhang F., Berg M., Dierking K., Félix M.A., Shapira M., Samuel B.S., Schulenburg H. (2017). *Caenorhabditis elegans* as a Model for Microbiome Research. Front. Microbiol..

[bib55] Zimmermann J., Obeng N., Yang W., Pees B., Petersen C., Waschina S., Kissoyan K.A., Aidley J., Hoeppner M.P., Bunk B. (2019). The functional repertoire encoded within the native microbiome of the model nematode Caenorhabditis elegans. bioRxiv.

